# A dynamic single cell-based framework for digital twins to prioritize disease genes and drug targets

**DOI:** 10.1186/s13073-022-01048-4

**Published:** 2022-05-06

**Authors:** Xinxiu Li, Eun Jung Lee, Sandra Lilja, Joseph Loscalzo, Samuel Schäfer, Martin Smelik, Maria Regina Strobl, Oleg Sysoev, Hui Wang, Huan Zhang, Yelin Zhao, Danuta R. Gawel, Barbara Bohle, Mikael Benson

**Affiliations:** 1 Centre for Personalized Medicine, Linköping University, Linköping, Sweden; 2 Department of Otorhinolaryngology, Yonsei University Wonju College of Medicine, Wonju, Korea; 3 Department of Medicine, Brigham and Women’s Hospital and Harvard Medical School, Boston, MA USA; 4 Channing Division of Network Medicine, Brigham and Women’s Hospital, Harvard Medical School, Boston, MA USA; 5 Department of Pathophysiology and Allergy Research, Center for Pathophysiology, Infectiology and Immunology, Medical University of Vienna, Vienna, Austria; 6 Division of Statistics and Machine Learning, Department of Computer and Information Science, Linkoping University, Linköping, Sweden; 7 Jiangsu Key Laboratory of Immunity and Metabolism, Department of Pathogenic Biology and Immunology, Xuzhou Medical University, Xuzhou, Jiangsu China; 8 Crown Princess Victoria Children’s Hospital, Linköping University Hospital, Linköping, Sweden; 9 Division of ENT Diseases, Department of Clinical Sciences, Intervention and Technology, Karolinska Institutet, Stockholm, Sweden

**Keywords:** ScRNA-seq, Inflammatory diseases, Upstream regulators, Multicellular network models

## Abstract

**Background:**

Medical digital twins are computational disease models for drug discovery and treatment. Unresolved problems include how to organize and prioritize between disease-associated changes in digital twins, on cellulome- and genome-wide scales. We present a dynamic framework that can be used to model such changes and thereby prioritize upstream regulators (URs) for biomarker- and drug discovery.

**Methods:**

We started with seasonal allergic rhinitis (SAR) as a disease model, by analyses of in vitro allergen-stimulated peripheral blood mononuclear cells (PBMC) from SAR patients. Time-series a single-cell RNA-sequencing (scRNA-seq) data of these cells were used to construct multicellular network models (MNMs) at each time point of molecular interactions between cell types. We hypothesized that predicted molecular interactions between cell types in the MNMs could be traced to find an UR gene, at an early time point. We performed bioinformatic and functional studies of the MNMs to develop a scalable framework to prioritize UR genes. This framework was tested on a single-cell and bulk-profiling data from SAR and other inflammatory diseases.

**Results:**

Our scRNA-seq-based time-series MNMs of SAR showed thousands of differentially expressed genes (DEGs) across multiple cell types, which varied between time points. Instead of a single-UR gene in each MNM, we found multiple URs dispersed across the cell types. Thus, at each time point, the MNMs formed multi-directional networks. The absence of linear hierarchies and time-dependent variations in MNMs complicated the prioritization of URs. For example, the expression and functions of Th2 cytokines, which are approved drug targets in allergies, varied across cell types, and time points. Our analyses of bulk- and single-cell data from other inflammatory diseases also revealed multi-directional networks that showed stage-dependent variations. We therefore developed a quantitative approach to prioritize URs: we ranked the URs based on their predicted effects on downstream target cells. Experimental and bioinformatic analyses supported that this kind of ranking is a tractable approach for prioritizing URs.

**Conclusions:**

We present a scalable framework for modeling dynamic changes in digital twins, on cellulome- and genome-wide scales, to prioritize UR genes for biomarker and drug discovery.

**Supplementary Information:**

The online version contains supplementary material available at 10.1186/s13073-022-01048-4.

## Background

Characterization and prioritization of pathogenic mechanisms in complex diseases, such as allergies and autoimmunity, are challenging because each disease may involve altered expression of thousands of genes across multiple cell types. On top of this complexity, those alterations may differ across different between time points of a disease process. The extent of such dynamic differences, on genome- and cellulome-wide scales, is largely unknown. This is an important explanation as to why medication is ineffective in some 40~70% of patients with complex diseases [1]. Digital twins are a promising concept first developed in engineering with the aim of computationally modeling complex systems such as airplanes or cities. They help to develop such systems, as well as predict and prevent malfunction, more efficiently than would be possible in real-life situations [2]. The medical counterpart, digital twins of patients, has been proposed as a solution for integrating the wide range of data relevant for human diseases, in order to improve prediction, prevention, and treatment [3, 4]. Current examples include the artificial lung, which models lung function based on ventilator measurements, and the artificial pancreas, which optimizes insulin treatment for type 1 diabetes patients based on continuous blood glucose measurements [5, 6]. These early, straightforward examples indicate how physiological and molecular variables can be used as starting points to construct digital twins. However, solutions for characterizing, organizing and prioritizing molecular changes on dynamic cellulome- and genome-wide scales are needed for diagnostic and therapeutic purposes [7]. Currently, the construction of digital twins of such complexity is considered intractable [4] as doing so would require solutions for a wide range of problems:

1. Characterization of disease-associated changes on dynamic cellulome- and genome-wide scales. Several studies of complex and malignant diseases indicate that this problem can be solved by genome-wide analyses, like single-cell RNA-sequencing. It can, however, also be complicated by difficulties in obtaining samples from relevant organs in human diseases, particularly if time-series analyses are required [8].
2. Organization and prioritization of scRNA-seq data are great challenges because of the large number of differentially expressed genes across multiple cell types. An ideal solution would be to identify an upstream regulator (UR) in one cell type that activates downstream genes in other cell types. Examples of such genes include IL4 in allergy and TNF in autoimmune diseases. Decades of research have shown that these are key regulators of important downstream pathways. This concept has, in turn, led to approved drugs targeting these UR genes [9]. However, these drugs show considerable variations in efficacy [10]. Currently, there is limited understanding of the reasons for such variation, and a lack of diagnostic tools to predict which patients will respond to a given treatment [11–13]. The reasons for varying efficacy are, therefore, not clearly defined, which raises an important question: to what extent have these URs and their downstream genes been studied in relation to the large number of other differentially expressed genes (DEGs) in each disease? The importance lies in the possibility that an UR could be co-regulated by other genes in multi-directional rather than linear hierarchies. There could also be other URs that are equally or more important. In clinical contexts, URs have been empirically prioritized and therapeutically targeted in scRNA-seq studies of immunological and malignant diseases [11]. However, the systematic prioritization of URs in human diseases, on cellulome- and genome-wide scales, is a key unresolved challenges. One possible solution would be to construct multicellular network models (MNMs) of diseases based on scRNA-seq data [14–16].
3. Such MNMs show predicted directed molecular interactions between cell types. Briefly, such interactions are predicted by bioinformatically inferring the URs of genes that are differentially expressed in a cell type. If that UR is expressed in another cell type, a directed interaction is predicted between the cell type harboring the URs and the cell type with the DEGs. Ideally, these interactions can be traced to one UR gene in each disease. To our knowledge, this issue has not been systematically investigated in human diseases. However, a recent study of a mouse model of arthritis found no single UR gene in an MNM derived from scRNA-seq data. Instead, the MNM formed a multi-directional network without any linear hierarchy. This observation led to application of network tools to prioritize cell types. Next, a gene module was identified in one of the prioritized cell types and therapeutically targeted. Limitations of the study included that UR genes were not systematically characterized and that the MNM was based on scRNA-seq data from one time point during symptomatic disease. These data could represent late-stage changes, which were induced by an UR gene only active at an earlier, pre-symptomatic stage. We have previously found such linear hierarchy in CD4 + T cells in immunological diseases [17].
4. Complex diseases may evolve over long periods, ranging from years to decades, before patients become symptomatic and receive diagnoses. Such processes may involve great molecular variations, which could be downstream of an early UR gene. If so, time-series analysis, ideally starting before the overt disease process, could reveal the UR in question. However, time series analyses are practically or ethically difficult in animal models and patients.

Here, we aimed to address these challenges using time-series scRNA-seq analysis of allergen-challenged PBMC from patients with seasonal allergic rhinitis (SAR). This approach may be optimal for modeling the dynamics of a complex disease process because the environmental trigger (pollen allergens) is known and absent outside of the pollen season when the patients are asymptomatic. Thus, the specific response process can be modelled in vitro by stimulating PBMC from SAR patients with a standardized dose of allergen outside of the pollen season. The process can be studied using time-series analyses before and during stimulation. The allergen is thought to induce a linear sequence of cellular events, in which activation of type 2 T helper (Th2) cells plays a key role [18]. This results in the release of the Th2 cytokines, IL-4, IL-5, and IL-13, all of which are approved or candidate drug targets in allergic diseases [18]. We constructed MNMs based on time-series scRNA-seq analyses of allergen/diluent stimulated PBMC from SAR patients and controls. Instead of a linear hierarchy, each MNM showed complex, multi-directional interactions between all the cell types, even before allergen-stimulation. Therefore, no single UR cell type or gene could be identified at any of the time points. This conclusion was supported by multicellular dispersion of the Th2 cytokines as well as by the fact that blocking one Th2 cytokine had no effect on the levels of other Th2 cytokines. Further analyses of bulk and single-cell data from allergic and inflammatory diseases in both lesional and non-lesional states revealed multi-directional networks without linear hierarchies, affirming this SAR-based observation and supporting its broader application. In the absence of linear hierarchies, we tried a quantitative approach to prioritize URs: we ranked the URs based on their predicted effects on downstream target cells. Experimental and bioinformatics analyses supported that such ranking is a tractable approach for prioritizing URs, and, thereby, for identifying potential biomarkers and drug targets in complex diseases. We propose that time series MNMs provide a scalable strategy for modeling and analyzing the dynamics of cellulome- and genome-wide changes in digital twins.

## Methods

### Study design

**Fig. 1:**
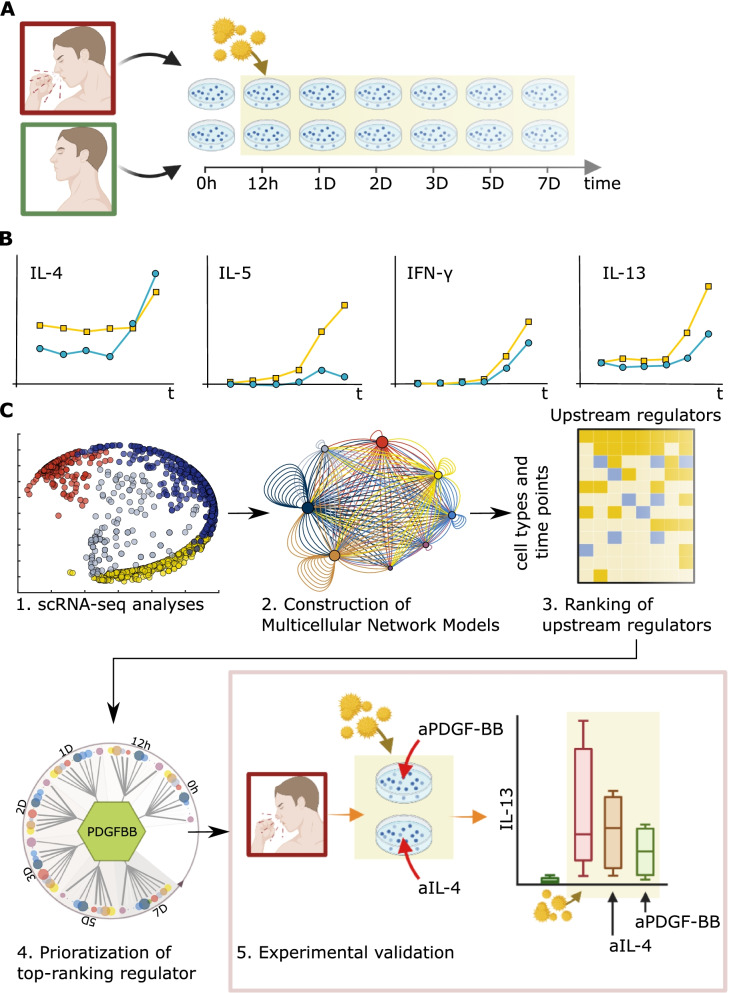
Overview of the study. **A** Stimulation of PBMCs from SAR patients (red) and non-allergic controls (green) with allergen or diluent. **B** Key Th1/Th2 cytokines (IFN-γ, IL-4, IL-5, and IL-13) were measured in supernatants from SAR patients (yellow) and non-allergic controls (blue) over time. **C** (1) Time-series scRNA-seq of PBMC from [19]. (2) Construction of MNMs, showing predicted molecular interactions between cell types at each time point. (3) Ranking of URs by the number of cell types that each is predicted to regulate at different time points (see the “Materials and Methods” section and Fig. 6). (4) Prioritization of the top-ranking UR that regulates the greatest number of the cell types at the greatest number of time points—platelet-derived growth factor B (*PDGFB*). (5) Validation studies, in which two URs, *IL4* and *PDGFB*, were blocked by specific antibodies

In summary, this study describes a scalable framework for inferring UR genes on dynamic cellulome- and genome-wide scales (Fig. 1). This framework is based on time series scRNA-seq analyses of allergen/diluent stimulated peripheral blood mononuclear cells (PBMC) from patients with SAR and healthy controls. We hypothesized that UR genes would be found at early time points. In order to identify URs, the scRNA-seq data were organized into directed MNMs of the different time points. We reasoned that the predicted directed molecular interactions in the early MNMs could be traced to a UR gene. However, no linear hierarchies could be identified in the MNMs, even before stimulation. Instead, our analyses of single-cell and bulk profiling data from allergic and other inflammatory diseases supported a quantitative approach to prioritizing UR, which was based on their predicted effects on downstream target cells.

### Participants

We included sixteen patients with SAR and fourteen matched healthy controls, from whom samples were obtained outside of the pollen season when both patients and healthy controls were asymptomatic. Of these, samples from fourteen controls and eight SAR patients were collected in Linköping, Sweden, and the remainder in Vienna, Austria. The samples from Sweden were used for isolating and in vitro culture of PBMC for scRNA-seq. Also, sera and supernatants were used to determine cytokine protein expression levels. The eight remaining samples, which were collected in Vienna, were used for blocking validation studies (Additional file 1). The median (range) ages of the patients were 29.5 (23~59) and 29.5 (21~39) for the healthy controls (Additional file 1). The Swedish samples from SAR patients were obtained for research purposes at the Department of Clinical Sciences, Linköping University. The patients from Vienna were also collected for research purposes at the Department of Pathophysiology and Allergy Research, Medical University of Vienna. The control samples were obtained from the donated blood at the Blood Donor Central at Linköping University Hospital. The inclusion criteria for SAR patients were a positive history for birch- or grass-pollen, or both pollens, inducing allergic rhinitis for at least 2 years. The sensitivity to birch or grass pollen was confirmed with skin prick tests (ALK Abello, Hørsholm, Denmark) and by an ImmunoCap Rapid Test (Phadia, Thermo Fisher Scientific, Uppsala, Sweden), which tests for birch, grass, and house dust mite sensitivity in all subjects. Exclusion criteria were positive IgE responses to house dust mites as well as any other diseases, including malignancies, diabetes mellitus, infectious diseases, severe deviation of the nasal septum, or nasal polyps. Written, informed consent was obtained from all participants, and the study was approved by the ethics committees of the universities of Linköping and Vienna.

### In vitro culture of allergen-challenge of PBMCs

PBMCs were enriched from buffy coats from healthy controls and fresh peripheral blood using Lymphoprep (Axis-shield, UK) with density centrifugation. PBMCs were incubated with birch pollen extract (BPE) (ALK Abello, 10 𝜇g/mL) at a density of 10^6^ cells/mL for different time periods in RPMI 1640 supplemented with 10% fetal bovine serum (FBS) (Gibco, USA). Before obtaining the cells, all sera were collected. The sera were aliquoted, frozen, and stored at −70 °C. All supernatants were collected after 12 h, 1 day, 2 days, 3 days, 5 days, and 7 days, respectively, and aliquoted, frozen and stored at −70 °C.

### scRNA-seq wet lab protocol

The scRNA-seq experiments for the samples from SAR patients and controls were performed using the Seq-Well technique [19]. In short, single-cell suspensions were prepared from cultured cells or fresh blood at each time point using standard techniques. Cells were counted and co-loaded with barcoded and functionalized oligo-dT beads (Chemgenes, Wilmington, Massachusetts, USA, cat. no. MACOSKO-2011-10) on microwell arrays synthesized as described with minor changes [19, 20]. For each sample, 20,000 live cells and ~110,000 beads were loaded per array. The cells and beads co-loaded in the microwell array were covered with previously plasma-treated polycarbonate membranes. Next, the array with the cover membrane was placed in a shaker at 37 °C for 45 min. After the membrane was removed, cell lysis and hybridization bead removal, reverse transcription, and whole transcriptome amplification were performed. Libraries were prepared for each sample using the Nextera XT DNA Library Preparation Kit (Illumina, San Diego, USA, cat. no. FC-131-1096) according to the manufacturer’s instructions. Libraries from three samples were pooled together for sequencing using the NextSeq 500/550 system, and sequencing results were analyzed as described below.

### scRNA-seq data processing

The single-cell data were processed into digital gene expression matrices following James Nemeshof Harvard Medical School’s McCarrol Lab’s Drop-seq Core Computational Protocol (version 1.0.1, Drop-Seq tools v1.12) [21, 22] using bcl2fastq (v2.19.1) conversion [23] and Picard software (v2.9.0) [24]. The indexed reference for alignment of the reads was generated from GRCh38 (April 2017, Ensembl) [25] using STAR software (v2.5.3) [26]. Only primary alignments towards the reference genome were considered during downstream analyses, according to the mapping quality using STAR software. The quality of cells was assessed by having a minimum of 10,000 reads, 400 transcripts, 200 genes, and less than 20% mitochondrial genes per cell. Outliers were removed based on empirical evaluation of the distribution of transcripts count over the cells, i.e., all cells with > 16,000 transcripts, due to the risk of duplicates in the library resulting in two or more cells sharing a cell barcode [27]. This processing resulted in a total of 9031, 8873, and 2871 cells for the non-stimulated, allergen-stimulated, and uncultured control, respectively, from the healthy individuals, and 5755, 6124, and 2244 cells for the non-stimulated, allergen-stimulated, and uncultured control, respectively, from the allergic individuals. To reduce the noise in the data, k-nearest neighbor (KNN)-smoothing with Python (v3.7.4) [28] was applied using a k of ~0.1% of the total number of cells (i.e., *k* = 14 and *k* = 21 for the data from allergic and healthy individuals, respectively) [29]. This proportional cut-off was selected to ensure that between-cell differences are maintained, but also to reduce noise optimally. The percentage 0.1% was selected based on expected cell type’s percentages among PBMC in healthy individuals [30].

### Microarray data processing (reference datasets)

To define cell types in the scRNA-seq data as described below, reference microarray data from B cells, CD4+, CD8+, monocytes, natural killer (NK) cells, naïve T cells, PBMC, T helper (Th)1, T helper (Th)17, T helper (Th)2, and T regulatory (Treg) cells were processed and analyzed [20]. Briefly, all microarrays were normalized using LIMMA R-package (version 3.32.10, R version 3.4) [31, 32]. We performed background correction using background Correct function with method “normexp,” followed by between arrays quantile normalization (normalize Between Arrays, method “quantile”). All probes with an expression below 1.2 times the background signal were removed.

### Cell type identification

To cluster the cells and define the cell types, reference component analysis (RCA) (v1.0) was performed [33] in R (v3.4) [32], for the healthy and patient group separately. The cells were projected against reference bulk-profiling data in a stepwise manner using different references for deeper subtyping in each step. First, cells were identified as monocytes, dendritic cells, B cells, or T/NK cells. For this step, the reference was constructed based on the HG_U133A/GNF1H (Affymetrix) gene atlas data set [34, 35], including only the cell types of interest, as described in the original paper [33]. In short, all genes with log10 (fold change) expression values greater than or equal to 0.5, relative to the median across all samples, were included. The resulting reference contained 746 genes (Additional file 2). T/NK cells were then further divided into CD4+ T cells, CD8+ T cells, and NK cells. Thereafter, CD4+ T cells were divided into their subtypes: Th1-, Th2-, Th17-, and regulatory T cells. For these steps, the references were constructed based on the microarray data from [20], including only the cell types of interest in each of the references. Here, all genes with log10 (fold change) expression values greater than or equal to 0.3 and 1, relative to the median across all samples, were included, resulting in references containing 1494 and 316 genes for the T/NK and CD4+ T cell subtyping, respectively (Additional file 2). The different log10 (fold change) cut-offs used to construct these references were based on empirical evaluations of the resulting clusters, where the aim was to obtain a clear separation of the cell types with a minimum number of cells of unclear identity.

During each step in the clustering and cell typing procedure, we also saved the Pearson correlation P-value for each cell from the RCA algorithm. With the aim to ensure credible cell types in the data, we removed the cells that did not match any cluster (*P*-value > 0.05 for all cell types in the reference), as previously described [20]. This filtering process resulted in a loss of ~12% of the cells (2345 cells from the healthy samples and 1839 cells from the allergic samples).

The clustering is presented in Fig. 3A and commented on in Additional file 3: Supplementary note 1.

### Global transcriptomic changes analysis

Global transcriptomic changes between time points for each cell type separately were assessed with a Euclidian distance-based method, as described [36].

For each cell type separately, we calculated average expression over all cells of the same type at the same time point, generating a library of representative cells (e.g., a representative cell of monocytes at day 1 was represented as a vector of the same length as the number of expressed genes, where the ith value within that vector was an average expression of the ith gene overall monocytes recovered at day 1). Next, we calculated the pairwise Euclidian distance between representative cells of the cell type between time points (e.g., monocytes at day 1 and monocytes at day 2). This distance was compared to a random distance calculated based on 1000 permutations. In each iteration time point labels were shuffled between cells of the same type (e.g., a monocyte recovered at day 1 might have been assigned as a monocyte at day 3 during a particular permutation), followed by calculation of the random representative cell type at the time point. The distance calculated between two different time points for each cell type was then compared to the null distribution of distances obtained from permutations.

### Differentially expressed genes

For single-cell data, DEGs were identified between allergen stimulated and diluent stimulated patients for each time point using Monocle (version 2.6.41) [37, 38] in R (v3.4) [32], as previously described [20]. When setting up the data for analysis, using newCellDataSet() function, a negative binomial distribution was defined (expressionFamily=negbinomial) and the lowest detection limit was set as lowerDetectionLimit= 0.5. Genes detected in at least three cells within a group were included in differential expression analysis using the differentialGeneTest() function. Genes were considered as significantly differentially expressed if the q-value < 0.05. Additionally, DEGs were calculated between allergen-stimulated allergic and healthy individuals, for each time point, and between uncultured control samples from allergic and healthy individuals.

### Identification of pathways, URs, and construction of MNMs

Pathways and URs were identified using the ingenuity pathway analysis (IPA) software (2019Q4-2021Q1) from January 19 to May 2021 [39]. Specifically, pathways in DEGs from bulk and single-cell data were identified using Canonical Pathways of Core analysis in IPA. To construct MNM at each time point, we started by identifying disease-associated genes (i.e., DEGs between allergen stimulated cells in patients and healthy controls) using the methods described above. If >5000 DEGs were found between two groups, the top 5000 DEGs (lowest q-values) were used for the IPA analysis, due to limitations of size allowance in IPA. Using those gene lists, MNMs were constructed: The Upstream Analysis of IPA software was queried for prediction of the URs of cell type-specific DEGs for each cell type at each time point separately. If such an UR was found in another cell type, a directed interaction between the two cell types was inferred. This analysis was performed at each time point separately. Here, we focused on URs that were secreted or membrane bound. If such an UR was found, an interaction was assumed between the cell types. For example, *PDGFB* was a predicted UR of DEGs in all cell types at day 2. *PDGFB* was identified as a DEG in monocytes. This observation led to the identification of a potential directed interaction from monocytes to all other cell types at day 2 (Fig. 7C). To reduce complexity, we did not incorporate potential autocrine interactions between monocytes.

### Potential drug identification

All possible drugs targeting DEGs at different time points in all cell types separately were identified with Molecules of Core analysis in IPA. Top 5000 DEGs were used for the IPA analysis, due to limitations of size allowance in IPA.

### Identification and ranking of the most important predicted URs or pathways

First, we analysed the top 5000 DEGs in all cells at all time points by IPA (version 2019Q4-2020Q1) from January 19 to May 2021 [31]. We identified all predicted URs and retrieved the lists of URs of each cell type, which were selected according to the following criteria: *P*-value <0.05 and |z-score| ≥2. Next, we counted the number of occurrences for each significant UR of each cell type at each time point. Finally, we ranked all significant URs, or pathways, based on the number of occurrences. We repeated the same analyses for enriched pathways of each cell type at each time point.

### In vitro validation assays

For blocking experiments, PBMCs (1 × 10^6^ cells) from eight SAR patients were stimulated for 5 days with 10 μg/mL BPE in the absence or presence of either a neutralizing anti-human IL-4 antibody (MAB204) or anti-human PDGF-BB antibody (AF-220-NA, both from R&D Systems, Minneapolis, USA) at a concentration of 5 μg/mL. Cell culture supernatants were collected at day 5 and the levels of IL-6, IL-13, and VEGF were measured by Luminex technology on a Bio-Plex 200, according to the manufacturer’s specifications (Bio-Rad Laboratories, California, USA). After collection of cell culture supernatants, PBMCs (2 × 10^5^ cells) were transferred to a 96-well plate and after another 16 h, T-cell proliferation was evaluated using incorporation of tritiated thymidine. Stimulation indices (SI) were calculated by dividing counts per minute (cpm) measured in stimulated cultures by cpm in cultures without stimuli.

### Validation experiment using human magnetic multiplex beads assay

The detection sensitivity for the protein and cytokine assays were CCL5 1.8 pg/mL; IL-4, 9.3 pg/mL; GM-CSF (CSF2): 4.1 pg/mL; IFN-α: 0.26 pg/mL; IFN-γ: 0.4 pg/mL; IL1RN, 18.0 pg/mL; IL-5, 0.5 pg/mL; IL-13, 36.6 pg/mL; PDGF-BB, 0.2 pg/mL. We used a five-parameter logistic curve to get the standard curve. Any sample that fell outside the recovery range (70~130%) in the curve area was considered inaccurate. If protein values lay outside of the detection limit for calculations, we assumed they were either the highest detection limit or a value of zero. We then performed a double-sided Wilcoxon rank sum test, by IBM SPSS statistics version 26, to compare protein concentrations in serum and supernatant between patients and healthy controls.

### Other datasets information

In this study, we used two bulk profiling datasets and two scRNA-seq datasets, which included inflammatory diseases, namely atopic dermatitis (AD), ulcerative colitis (UC), and Crohn’s disease (CD). The bulk microarrays were used to analyze the gene expression in endoscopic-derived intestinal mucosal biopsies from patients with inflammatory bowel diseases (UC and CD) and healthy controls, and intestinal mucosal biopsies included both lesional and non-lesional gut mucosa in GSE75214 [40] (in total 97 UC patients, eight CD patients, and 11 controls). GSE32924 [41] included paired samples of both lesional and non-lesional skin from 12 patients with AD compared with normal human skin from eight healthy controls [41]. Using GEO2R [42] with default settings, we identified DEGs between lesional and healthy control samples, as well as between non-lesional and healthy controls, in the gut or skin, from patients with UC, CD, or AD. The data were annotated using the National Center for Biotechnology Information (NCBI) generated platform [43]. They were then adjusted for multiple testing using the Benjamini-Hochberg procedure [44]. The data were then sorted to only include significant DEGs (FDR < 0.05) for downstream analysis, which included GWAS gene enrichments. Genome-Wide Association Studies (GWAS) genes were obtained from DisGeNET [45] (data downloaded on Feb 9, 2021). In total, 416 GWAS genes for CD, 359 genes for UC, and 126 genes for AD were retrieved. Enrichment analyses of GWAS genes respective DEGs were performed using Fisher’s exact test. All genes from respective datasets were used as background for the enrichment analysis.

URs were identified using The Upstream Analysis of IPA*.* Significant URs were selected based on |z-score|≥2 and overlap *P*-value < 0.05. Similarly, all drugs with possible effects on DEGs were identified using Molecules of Core analysis in IPA as described above. To view how DEGs were predicted to interact with each other in AD (GSE32924 [41]), we created a gene network using Networks of Core analysis in IPA. First, Molecules tool was used to identify all possible networks representing interactions between single UR and a single-downstream effect (function/disease) and the dataset genes involved in both. The top five networks with the highest consistency score were merged and visualized as a graph.

The scRNA-seq data for UC included 12 colon biopsies from five patients including four non-inflamed and four inflamed biopsies as well as four healthy individuals [46]. The DEGs between biopsies from non-inflamed tissues from patients and healthy controls, as well as between inflamed biopsies from patients and healthy controls were downloaded from [46] and used for further downstream analyses. The scRNA-seq data for CD (E-MTAB-8901) included ileum biopsies from seven patients and eight healthy individuals, where five samples were from inflamed tissue, two samples from non-inflamed tissue, and eight samples from healthy control tissue [47]. Using the processed data, with cell types as identified in [47], we identified DEGs between non-inflamed and healthy ileums, as well as between inflamed and healthy ileums, using Monocle as described under the “Differentially expressed genes” section above.

## Results

### Allergen-stimulation induces a Th2-like response in PBMC from SAR patients

**Fig. 2:**
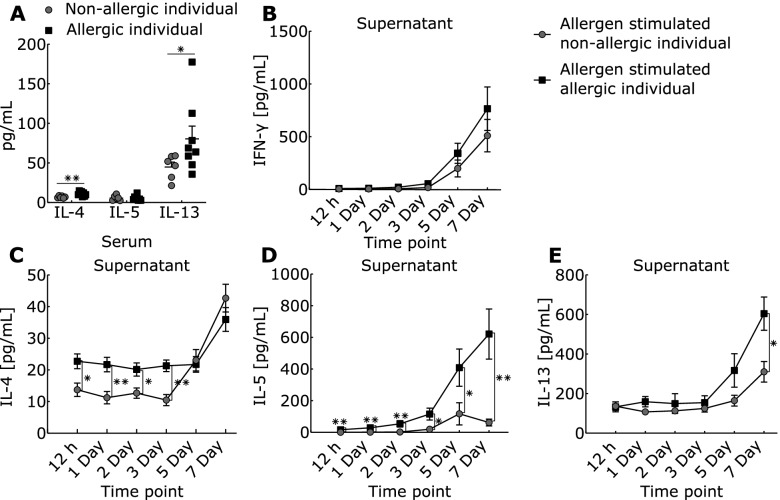
Th1/2cytokine levels in supernatants from allergen-stimulated PBMC and sera from healthy controls and allergic patients. **A** IL-4, IL-5, and IL-13 levels in sera. **B** IFN-γ, **C** IL-4, **D** IL-5, and **E** IL-13 levels in supernatants. *P-value < 0.05, **P-value < 0.01, Wilcoxon signed rank test

To establish a complex disease model, we performed in vitro allergen-challenge of PBMCs from SAR patients and healthy controls [48]. Sera and supernatants were harvested before and after 12 h, as well as after 1, 2, 3, 5, and 7 days of incubation. Serum levels of IL-4 (*P*-value = 0.01) and IL-13 (*P*-value = 0.039), but not IL-5, were significantly higher in allergic patients than in healthy controls (Fig. 2). In supernatants of allergen-challenged PBMCs from allergic patients, IL-4 was significantly higher at early time points, while IL-5 and IL-13 increased at later time points (Fig. 2C, E). All Th2 cytokines showed considerable inter-individual differences between patients (Additional file 3: Fig. S1). In contrast to Th2 cytokines, no significant differences in the signature Th1 cytokine IFN-γ were found between SAR patients and controls (Fig. 2B). These data were in agreement with current understanding of allergic inflammation resulting from increased activity of Th2 cells relative to Th1 cells and thereby support our disease model [49].

### scRNA-seq shows great diversity of gene expression changes in SAR patients across time points and cell types

**Fig. 3:**
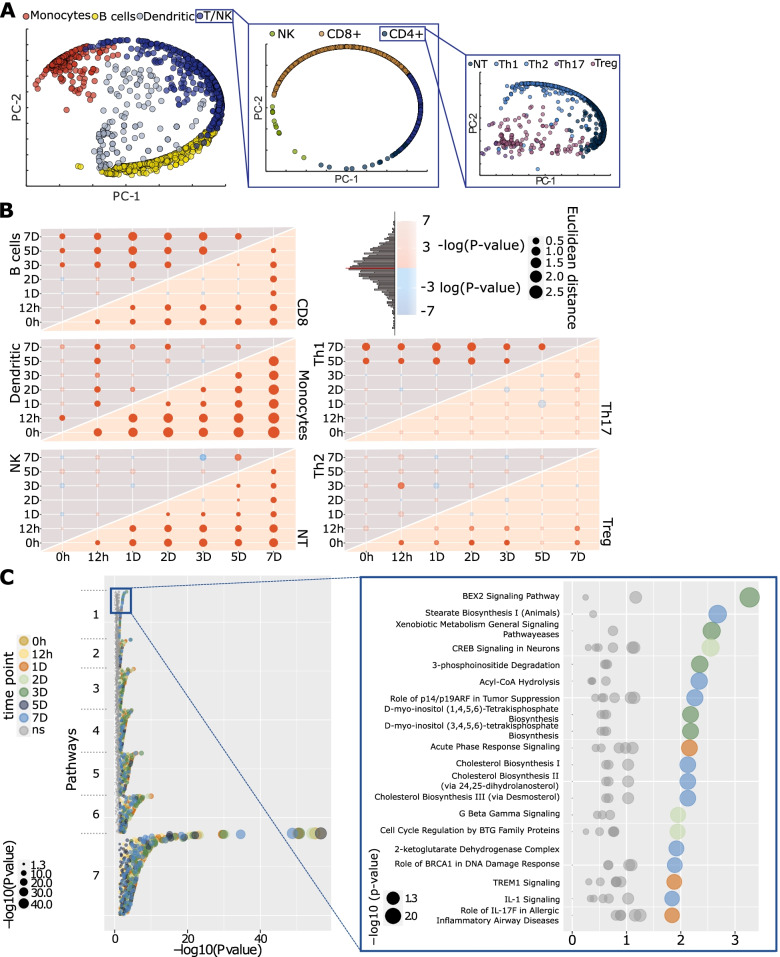
Diversity of gene expression across cell types and time points. **A** Principal component visualization of how each single-cell transcriptome correlated with cell type-specific bulk transcriptomes [33]. Only allergic patients’ cells stimulated with allergen are presented in the figure. Cells were classified into subsets in three main steps using RCA [33]. **B** Dot plot showing global transcriptomic shifts assessed with Euclidian distance (Ed) compared to a random distribution. Red and blue colors denote that empirical Ed was higher and lower than the random mean Ed, respectively. The size of the nodes represents empirical Ed. **C** Dot plot presenting all pathways enriched in DEGs at any time point in Treg cells. The colors of the dots represent different time points (not significant – ns, enrichments are shown with gray color), whereas their size denotes enrichment significance -log10(*P*-value)

The increase of Th2 cytokines in supernatants supported their potential UR roles in this in vitro model of allergic inflammation. However, previous studies by us and others have shown altered expression of thousands of genes in allergen-stimulated CD4 + T cells [49]. To assess the roles of Th2 cytokines relative to the many other DEGs, we performed Seq-Well-based massively parallel scRNA-seq on PBMC from three SAR patients and four healthy controls at all time points before and after allergen or diluent treatment. After filtering using a minimum of 10,000 reads and 400 transcripts per cell, we captured 34,897 cells after performing quality controls, with an average of 712 cells per individual and time point (Additional file 4). Cell type classification was performed using Reference Component Analysis (RCA, the “Materials and Methods” section ) [33]. In total, we identified ten cell types, or subsets thereof, namely B lymphocytes, monocytes, dendritic cells, CD8+ T lymphocytes, NK cells, naïve T (NT) cells, regulatory T (Treg) cells, Th1 cells, Th2 cells, and Th17 cells (Fig. 3A, Additional file 3: Fig. S2 and S3).

**Fig. 4:**
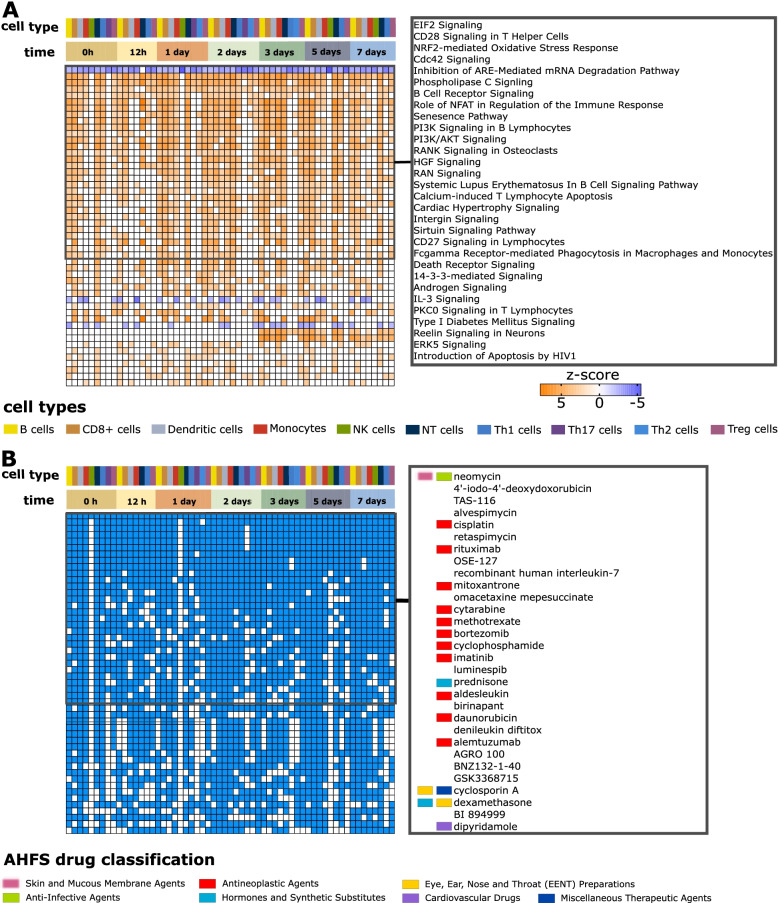
Ranking of pathways and therapeutic targets. **A** Heatmap of top 50 pathways ranked based on their enrichment in different cell types at different time points (|z-score|≥ 2 and *P-*value < 0.05). The color intensity of orange and blue boxes indicates significance of enrichment. Gray boxes indicate non-significant predictions. An orange box indicates directions of the differential expression that match the predicted direction, while a blue box indicates the opposite (z-score ≥2 or ≤−2, respectively). **B** Heatmap representing top 50 drugs predicted to target most of the cell types at most time points. A blue square means that the drug was predicted to target a cell type at the time point, and a white square denotes that the drug was not predicted to target a cell type at the time point. Drug names identified with IPA were matched with DrugBank drug names. For matching drugs American Hospital Formulary Service (AHFS) classification numbers were retrieved, and the overriding categories listed at RxID [50] are reported in the figure. The inserted boxes on the right correspond to the top 30 pathways (**A**) and drugs (**B**) in the left boxes

To assess systematically global transcriptome shifts triggered by allergen stimulation in cells derived from allergic patients, we performed Euclidian distance- and permutation-based analyses (Fig. 3B; the “Materials and Methods” section). In summary, these analyses showed that allergens stimulated a great diversity in transcriptomic shifts across time points, in which Th2 cytokines were not most differentially expressed: In short, for each cell type analyzed separately, we computed an average expression profile and analyzed how much each cell type’s expression profile differed from each other. Next, we performed differential gene expression analyses, comparing patients and controls at each time point following allergen-stimulation, and for each cell type separately (the “Materials and Methods” section). These DEGs reflected a great diversity of pathways across cell types and time points. The median number of pathways per cell type was 195 (0–288) (Fig. 3C, Additional file 5). Since this diversity was found even before allergen-stimulation, analysis of that time point did not indicate an early UR pathway that activated other pathways in a linear hierarchy (Fig. 4A). This complexity suggested that specific therapeutic targeting of the most significant pathway in one cell type would not suffice because of multiple other activated pathways in the same or other cell types. Indeed, the DEGs from the scRNA-seq data could be targeted by 1619 possible drugs (Fig. 4B). The median number of drugs targeting DEGs in any cell type at any time point was 231 (range: 1–671) (Additional file 6). Thus, an impractical number of drugs targeting multiple pathways might be needed. This cellular and molecular diversity was also found when specifically focusing on Th1 and Th2 cytokines. Although patients displayed Th2-like responses, and controls had Th1-like responses when comparing allergen- and diluent stimulated cells, these responses varied greatly across cell types and time points. A similar complexity was found in comparisons between allergen-stimulated cells from patients and controls (Additional file 7).

**Fig. 5:**
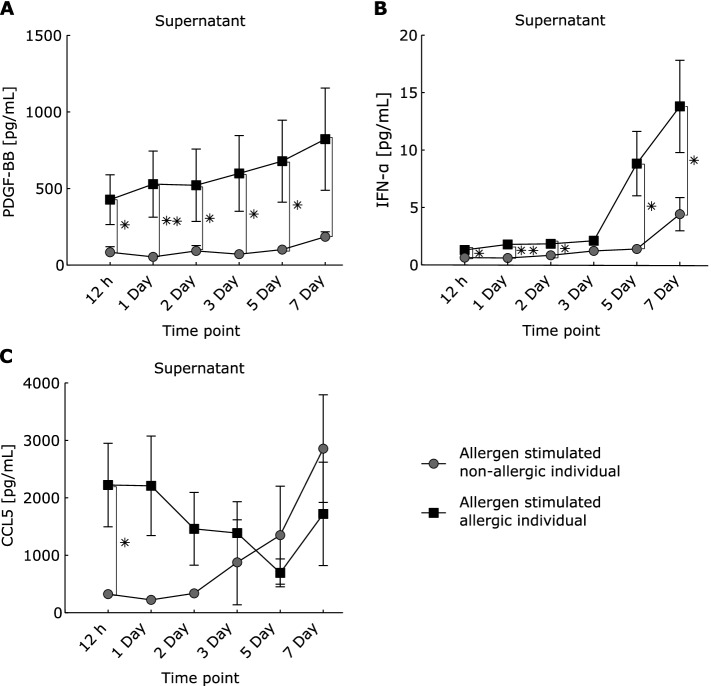
Protein expression patterns of predicted URs in supernatants of allergen-stimulated PBMC from SAR patients and controls. **A** PDGF-BB, **B** IFN-α, and **C** CCL5 levels in supernatants. *P-value < 0.05, **P-value < 0.01, Wilcoxon signed rank test

In order to assess systematically the UR roles of Th2 cytokines relative to other URs, we bioinformatically inferred all URs of the DEGs in each cell type. These URs were ranked based on the number of cell types and time points at which the URs had significant effects on the DEGs they were predicted to regulate (Additional file 8). These analyses were performed for DEGs derived from comparisons between allergen-stimulated cells from patients and controls, in all cell types at all time points. In other words, we searched for and prioritized the URs that differed the least between time points. Instead of Th2 cytokines, the top-ranking URs (rank number) were *PDGFB* (1), IFN alpha and beta receptor subunit 2 (*IFNAR2*) (2) and its ligand *IFNA2* (15), prolactin (*PRL*) (3), and C–C motif chemokine ligand 5 (*CCL5*) (14) (Additional file 8 and Fig. 7D)*.* All of these genes were predicted URs before allergen-stimulation, as well as most other time points. We next analyzed the protein products of the highest-ranking URs in supernatants from the eight SAR and six healthy control samples. We found significant increases of PDGF-BB and IFN-α at most time points, while CCL5 was only significant at 12 h and 1 day (Fig. 5).

**Fig. 6:**
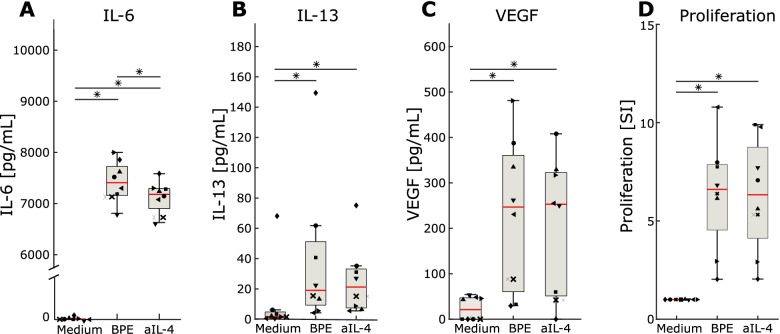
Effects of neutralization of IL-4 on IL-13, IL-6, and VEGF from allergen-stimulated PBMC from SAR patients. **A** IL-16, **B** IL-13, and **C** VEGF levels in supernatants harvested at day 5; **D** allergen-specific lymphoproliferation. Red lines in box plots indicate median values, *P-value < 0.05, Wilcoxon signed rank test. The different shapes of data points represent different SAR patients

To obtain a functional overview of the mechanisms regulated by the top-ranking URs, we performed pathway analysis of the DEGs that were predicted to be regulated by these URs. Pathway analysis of the putative PDGF-BB-regulated genes showed that these had highly pleiotropic effects of potential relevance for allergies: B cell signaling, cell migration and proliferation, immune response, and cytokine-related pathways, for example, via IL-6, IL-7, IL-8, and IL-15 signaling (Additional file 9). The corresponding analyses of IFN-α and CCL5-regulated DEGs also showed enrichment of cytokine-related pathways (Additional files 10 and 11). Interestingly, IL1 receptor antagonist (*IL1RN*), an anti-inflammatory gene, decreased at all time points in most cell types. This observation supports previous research pointing to IL1RN as a potential drug for treating allergies [51]. Taken together, these results showed multiple URs and pathways the functions of which varied greatly across cell types and time points. Collectively, the DEGs interacted in multi-directional networks, rather than linear, unidirectional hierarchies, between cell types. The highest ranking Th2 cytokine URs were *IL5* (rank number 13) and *IL4* (59). By contrast, *IL13* did not have significant predicted effects on downstream mRNA expression levels in any of the cell types (Additional file 8). This observation was consistent with the significant early increases of IL-4 and IL-5 protein levels, while IL-13 did not increase significantly until day 7 (Fig. 2), such that downstream effects of IL-13 could be delayed compared to IL-4 and IL-5. This delay also suggested a possible linear hierarchy in which IL-4 and/or IL-5 regulated IL-13. To test this possibility, we examined if neutralization of IL-4 in allergen-stimulated PBMC from SAR patients affected the levels of IL-13, and two other proteins predicted to be regulated by IL-4, namely IL-6 and vascular endothelial growth factor A (VEGFA). The addition of neutralizing anti-IL-4 (aIL-4) antibodies resulted in a significant decrease of IL-6, but not of IL-13 or VEGFA levels (Fig. 6A–C). To demonstrate that the observed effect was not due to a reduced T cell response to allergen, we assessed allergen-induced lymphoproliferation in the absence or presence of aIL-4. The proliferation of allergen-specific T cells was not affected by aIL-4 antibodies (Fig. 6D). Taken together, these analyses suggested that Th2 cytokines had variable regulatory roles relative to other URs and that there was no linear hierarchy between IL-4 and IL-13. Since in vitro stimulation may not be representative of the complex interactions between immune and stroma cells in allergic tissues, we assessed the in vivo relevance of our findings by comparisons with bulk profiling data from patients with AD [41]. None of the three Th2 cytokines were differentially expressed or predicted UR (Additional file 3: Fig. S4 and Additional file 12). Similar to the scRNA-seq data the DEGs formed a complex network in which no linear hierarchy was found (Additional file 3: Fig. S5). Pathway analysis of the bulk profiling data also showed a great diversity (Additional file 12).

### Time series MNMs support multidirectional interactions without linear hierarchies

**Fig. 7:**
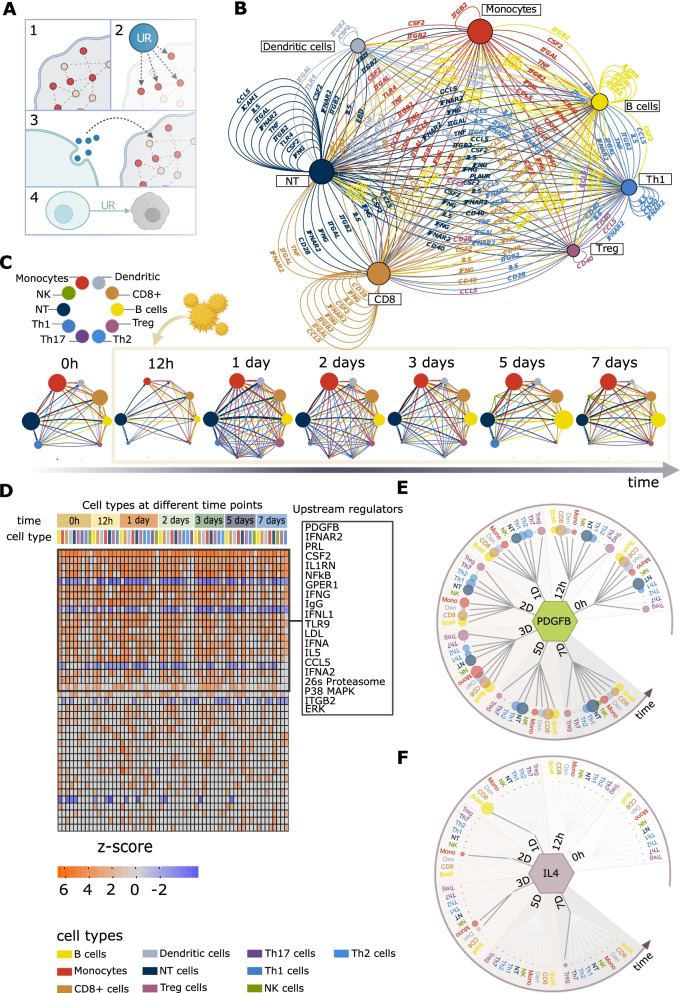
Construction of MNMs and prioritization of URs. **A** (1) Determination of DEGs (red) in a cell type (gray). (2) Bioinformatic identification of the predicted UR of the DEGs. (3) Identification of another cell type (blue) in which that UR is differentially expressed. (4) A directed interaction from the blue to the gray cell type is formed. **B** Example of one MNM (at 0 h) which was constructed based on directed interactions as described in A. **C** MNMs created at each time point. Node sizes correspond to the number of DEGs between cells isolated from patients compared to healthy controls. Edge width represents the number of predicted URs. Edge color corresponds to the source cell. **D** Heatmap of top 40 URs ranked based on the number of cell types that each UR is predicted to regulate at different time points. Color intensity boxes indicate the statistical significance of predictions. Grey boxes indicate non-significant predictions. A positive z-score indicates that the direction of the differential expression matches the predicted direction (orange), while a negative z-score indicates the opposite (blue). The inserted boxes on the right correspond to the top 20 URs in the left boxes. **E**, **F** Dynamic UR-target models showing predicted cell-type targets of PDGF-BB and IL-4 in allergen-stimulated patients. Node size denotes the significance of the association (z-score)

A limitation of the above analyses of molecular interactions is that they were performed in each cell type separately, without considering intercellular interactions. Thus, although we did not find that intracellular interactions were organized in linear hierarchies, it is possible that there could be such hierarchies between cell types. If so, the interactions might be traced to a UR gene, particularly at early time points at which the disease process was initiated. To test this possibility, we constructed multicellular network models (MNMs) of all the time points and found predicted directed molecular interactions between cell types. Yet again, however, none of the MNMs showed interactions that were organized in unidirectional linear hierarchies. The interactions were bioinformatically inferred by linking the DEGs in each cell type to their predicted UR in all other cell types (Fig. 7A) [39]. For example, *PDGFB* increased in monocytes at day 2 and was a predicted UR of DEGs in all cell types, which led to the identification of a directed interaction from monocytes to all other cell types at day 2 (Fig. 7A–C). However, all MNMs from allergen-stimulated PBMC from patients vs. controls, as well as between allergen vs. diluent stimulated PBMC in SAR patients and controls, were multi-directional without any evident linear hierarchies (Fig. 7B, C, Additional file 3: Fig. S6). Importantly, even before allergen-stimulation, a highly complex, multi-directional MNM was found. At each time point, we found a median of 10 (range 1–23) URs that were predicted to target a median of 2 (1–9) cell types.

### Ranking and prioritization of URs based on their downstream effects

Since no single-UR gene could be identified at any time point, we reasoned that a tractable strategy to prioritize URs would be to rank them based on their downstream effects. In short, the rank of each UR was inferred from the number of predicted target cell types with significant enrichment of downstream genes of that UR among DEGs at all time points (Fig. 7D–F, Additional file 8).

**Fig. 8:**
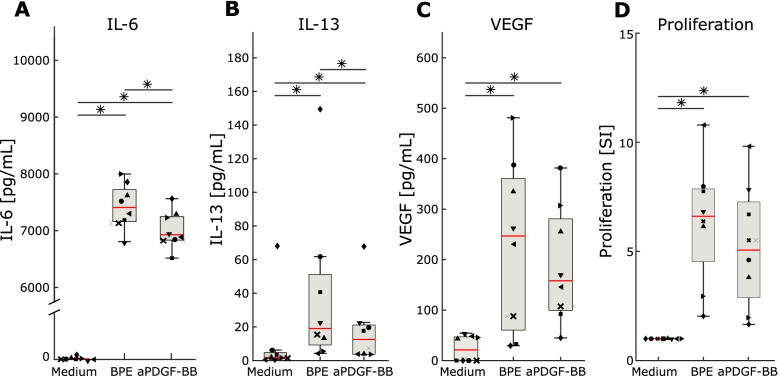
Effects of PDGF-BB neutralization on the release of IL-6, IL-13, and VEGF from allergen-stimulated PBMC. **A** IL-6, **B** IL-13, and **C** VEGF levels in supernatants harvested at day 5; **D** allergen-specific lymphoproliferation. Box plots are shown, red lines indicate median values, * *P*-value < 0.05, Wilcoxon signed ranks test. The different shapes of data points represent different SAR patients

We focused on *PDGFB* because it had the highest rank at all time points, even before allergen-stimulation; the pleiotropy of its downstream DEGs; and consistent increases of its protein product at all time points. We tested the effects of a neutralizing anti-PDGF-BB (aPDGF-BB) antibody on the release of IL-6, IL-13, and VEGF from allergen-stimulated PBMC. The analyses were performed on PBMC from eight different SAR patients, and the cytokines were selected because they are the predicted targets of PDGF-BB [52–55]. This stimulation resulted in significant decreases of IL-6 and IL-13 (Fig. 8A, B). We also observed a tendency for reduced VEGFA, however, without reaching statistical significance (Fig. 8C). Allergen-induced T cell proliferation was not affected (Fig. 8D).

Taken together, these analyses supported that URs could be ranked and prioritized, despite the large and time-dependent variations on cellulome- and genome-wide scales. However, the ranking strategy could be confounded by being derived from an in vitro model of only one disease. We therefore proceeded to test the strategy in in vivo data from other diseases.

### Meta-analysis of inflammatory diseases supports the importance of URs that are shared across time points and cell types

The time series scRNA-seq analysis of allergen-stimulated PBMC in SAR pointed to two systems-level factors complicating the prioritization of URs: multiple pathogenic mechanisms that (1) interact in multidirectional networks, rather than in linear hierarchies and (2) vary at different disease stages. To examine if these factors were generalizable to other diseases, we analyzed bulk and single-cell profiling data from non-lesional and lesional biopsies from three other diseases, AD, ulcerative colitis (UC), and Crohn’s disease (CD), as well as from healthy controls [40, 41, 46, 47]. We reasoned that the two types of biopsy specimens represented different stages of each disease, similar to PBMC before and after allergen-stimulation. Indeed, the bulk profiling data from each disease showed thousands of DEGs that differed greatly between the two states (Additional files 12, 13, and 14). These DEGs formed multi-directional networks that were enriched for multiple pathways and URs, which only partially overlapped (Additional file 3: Fig. S6). For example, in UC, there were 2889 DEGs in non-lesional and 9387 DEGs in lesional gut biopsies. These DEGs were the predicted targets of 206 and 708 drugs, respectively, of which 175 overlapped (Additional file 13). In support of the pharmacological relevance of prioritizing overlapping URs, *TNF* was a top-ranking UR in both non-lesional and lesional biopsies, which agrees with the known importance of TNF as a drug target; however, lesional gut biopsies also included other top-ranking URs like *IFNG*, *IL1B*, and colony-stimulating factor 2 (*CSF2*). All four URs, and the DEGs they were predicted to regulate, interacted in partially overlapping networks without any evident linear hierarchy. Moreover, there were 99 other URs in non-lesional and 315 URs in lesional gut in UC. A similar complexity was found in AD and CD (Additional files 12 and 14).

**Fig. 9:**
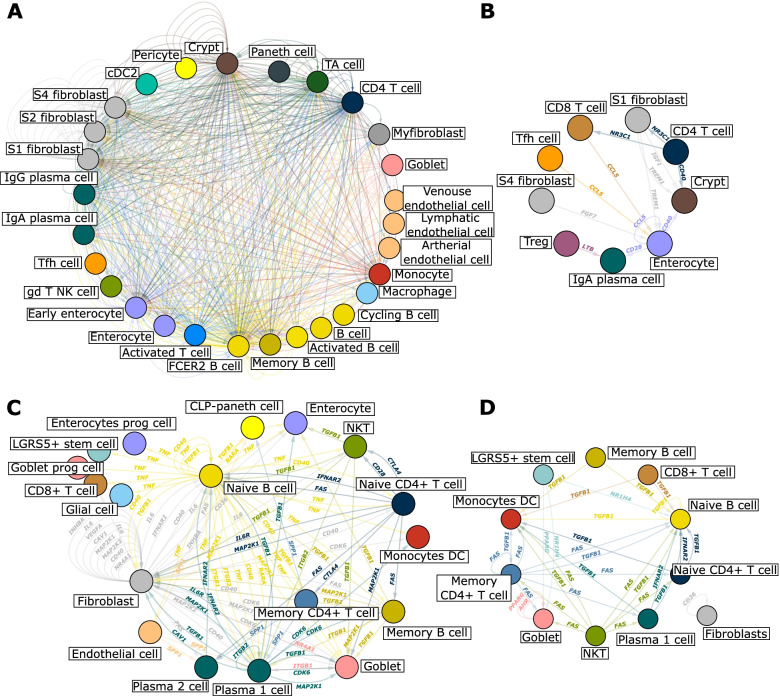
MNMs of different states of UC and CD. **A** Lesional state in CD, **B** non-lesional state in CD, **C** lesional state in UC, and **D** non-lesional state in UC

The absence of linear hierarchy could depend on the UR predictions being derived from bulk profiling data, which do not distinguish between molecules that interact within or between cells. To address this limitation, we constructed scRNA-seq-based MNMs from non-lesional and lesional gut biopsies from patients with CD and UC [46, 47]. Similar to MNMs from allergen-stimulated PBMC from SAR patients, we found multi-directional networks of great diversity between non-lesional and lesional biopsies (Fig. 9, Additional file 3: Fig. S5). In CD, the MNMs were the predicted targets of 643 and 2114 drugs in non-lesional and lesional biopsies (Additional file 15). The corresponding numbers for UC were 957 and 1359 drugs, respectively (Additional file 16). There were 135 and 668 predicted URs in non-lesional and lesional biopsies of CD as well as 167 and 325 in UC, respectively, of which 120 and 141 overlapped. Similar to the bulk-profiling data, *TNF* was the top-ranking UR in non-lesional and lesional UC. *TNF* was also top-ranking URs in CD, but not in non-lesional CD. The top-ranking URs in CD and UC included *PDGFB*, *IFNA2*, *IL1*, and *IFNG.* Taken together, both bulk and single-cell data indicated that prioritization of drug targets would be complicated by multiple URs, which differed between disease stages. We reasoned that the prioritization could be simplified by searching for DEGs and URs that were enriched for genetic variants identified by Genome-Wide Association Studies (GWAS). We identified GWAS genes from each disease using DisGeNET (data downloaded from February 9, 2021 version). We identified 416 genes for CD, 359 genes for UC, and 126 genes for AD. We applied Fisher’s exact test to test the enrichment of GWAS genes in DEGs. We found that DEGs from bulk-profiling data from UC and CD, but not AD, were enriched for GWAS genes. The enrichment was more significant in lesional than non-lesional states of UC and CD. The respective P-values for lesional and non-lesional states were 4.44 × 10^8^ and 0.028 for UC. The corresponding P-values for CD were 9.34 × 10^7^ and 0.039. The GWAS-enriched DEGs from lesional states were part of a wide variety of inflammatory, proliferative, and metabolic pathways, some of which were also significant in non-lesional UC (Additional file 17). Those overlapping pathways included IL-17 signaling (increased) and cholecystokinin/gastrin-mediated signaling (increased). Both pathways contain URs that are potential drug targets, namely *IL17A*, *IL17RA*, and *TNF*. In CD, the GWAS-enriched DEGs from the lesional biopsies were also components of a wide variety of pathways. The corresponding DEGs from non-lesional biopsies were not enriched for any pathways; however, one of the top URs, triggering receptor expressed on myeloid cells 1 (*TREM1)*, regulated one of the most significant GWAS-enriched pathways in lesional CD, namely TREM-1 signaling. TREM-1 is a major amplifier of innate immune responses and a drug candidate in inflammatory diseases [56].

## Discussion

Despite increasing numbers of drugs that precisely target genes considered URs, many patients do not respond adequately to drug treatment. One reason for this significant pharmacological shortcoming is the complexity of the cellulome*-* and genome-wide changes in many diseases. Other reasons are that diagnosis and treatment are often delayed because of the late occurrence of symptoms in disease processes that evolve over long periods. The aim of medical digital twins is to bridge this gap by providing dynamic multi-scale models of diseases or individual patients. Broadly speaking, a digital twin has been defined as an in silico model that brings together the technology to map, monitor, and control real-world entities by continually receiving and integrating data from the physical twin to provide an up-to-date digital representation of the physical entity [57].

Early examples, like the artificial pancreas, which is currently undergoing clinical trials [6], as well as methods to integrate multi-scale data, support the potential of digital twins [58]. However, limitations of these examples are that they neither show disease-associated changes on cellulome- and genome-wide scales, nor prioritize between such changes. This type of analysis of disease-associated changes and their prioritization is crucial for identification of the most relevant disease genes, as well as diagnostic and therapeutic targets [3]. In this study, we hypothesized that these problems could be resolved by time series scRNA-seq-based MNMs to trace UR cell types and genes. We found that these MNMs could model disease-associated gene expression changes across multiple cell types in four immunological diseases, as well as molecular interactions between those cell types. Because the interactions formed multidirectional networks, rather than linear hierarchies, tracing the molecular interactions to an UR cell type and gene was impossible in any of the diseases. Instead, the MNMs were regulated by multiple URs that could be ranked quantitatively based on their cellular and molecular effects. In agreement with the known efficacy of drugs targeting TNF in autoimmune diseases, this gene was among the top-ranking URs in both UC and CD. In SAR, IL-4 was a significant, but not a top-ranking UR. Together, these findings support that MNMs are applicable to model cellulome- and genome-wide changes in diseases and potentially to prioritize URs. We propose, therefore, using MNMs as a scalable approach to integrate such models in digital twins. However, our findings also indicate levels of complexity that challenge current paradigms for diagnostics and therapeutics. While such challenges may seem intractable, the suffering and costs associated with the large number of patients who do not respond to treatment emphasize the urgency in addressing systematically those challenges. Our strategy to identify and rank URs may be one solution. For example, in UC and CD, the top ranking URs included candidate drug targets other than *TNF*, such as *IL-1* and *IFNG*, suggesting that combinatorial treatments may be needed. As recently reviewed, such combinations may include biological drugs specifically targeting individual URs, more broadly acting immunosuppressive drugs, as well as new modalities like cellular therapies [59]. In addition to targeting one or more top-ranking URs, another solution for personalized treatment combinations could be to target DEGs that are enriched for GWAS genes. This goal could be met by targeting the URs of those DEGs or other broadly acting modalities targeting the DEGs. We found that lesional states of UC and CD were enriched for GWAS genes, which belonged to pathways with a wide range of inflammatory, metabolic, and cell proliferative functions. This pleiotropy is supported by meta-analyses of GWAS, which implicate large numbers of genes of low or moderate effects that collectively contribute to complex diseases [60–62]. Such meta-analyses are consistent with the notion that diseases reflect multiple perturbations of complex intracellular networks [63]. Although the complexity and stage-dependent variations of the molecular changes, as well as the lack of linear hierarchy, make drug target prioritization a formidable challenge, our study points to other possible solutions for further studies. Stage-dependent variations indicate that a drug may be effective at one time point, but not another. This conclusion was supported by our studies of SAR and AD, in which Th2 cytokines, which are known drug targets, had variable mRNA and protein expression levels, ranks, and downstream experimental effects. This problem may be resolved by time series analysis to prioritize and target URs that have high ranks across multiple time points. Indeed, top-ranking URs in SAR, like *PDGFB*, had consistent expression changes and downstream experimental effects. In UC, *TNF* had high ranks, which is consistent with drugs targeting TNF being extensively clinically used for autoimmune diseases. However, given the heterogeneity of the effects of top-ranking URs, it is likely that combinatorial treatments will be required.

An important limitation of this study is that it is restricted to cellular and molecular changes. Although such changes are primary targets of pharmacological interventions, construction, analyses, and clinical implementation of digital twins will require integration and analyses of multiple types of clinically relevant data [3, 4]. Other technical limitations include problems associated with processing of scRNA-seq data, such as drop-outs. We have attempted to address such issues as described and commented upon in Additional file 18. Inference of ligand-receptor interactions based on IPA could be confounded by knowledge-bias. IPA is a commercial software that is based on comprehensive mining of the medical literature. Other non-commercial tools have been recently described that may be less knowledge-biased [14–16] To compensate for this potential limitation, we performed functional and bioinformatics analyses, such as searching for GWAS enrichment. It should also be noted that IPA also includes data from multiple, less biased, databases. Another potential limitation is that samples from SAR and AD patients were taken from sensitized individuals, which means that early, undetected events that influence later events in linear hierarchies cannot be excluded. Such early events may be inferred using gene regulatory networks [17]. Another problem is that the complexity of scRNA-seq analyses with current technologies makes their use in clinical settings impractical. However, there are case reports of scRNA-seq-guided treatments of individual patients with immunological and malignant diseases [64, 47]. One solution could be to structure centralized analyses to facilitate clinical implementation of scRNA-seq analyses [3, 8, 48, 65]. In clinical settings, this may result in time-dependent personalized prescriptions of drug combinations, tailored to the time-varying disease state of an individual. Time-dependent prescriptions could be simplified by focusing on treating states in which MNMs are least complex. For example, in UC and CD, MNMs from non-lesional tissues were less complex than in lesional [3, 4]. Thus, biomarkers and drugs specifically targeting non-lesional MNMs or URs could be used during remission, to prevent relapse. Another option could be to develop drugs that convert lesional to non-lesional states in order to guide remission-inducing therapies. Despite these challenges, the medical and economic needs to improve treatment efficacy, as well as genomic and computational advances, may pave the way for digital twins that include MNMs for predictive, preventive, and personalized treatment. This will require construction of multi-scale digital twins, which allow automated drug response predictions, as well as tools to functionally understand those predictions. Given the complexity of those challenges, large-scale international collaborative efforts will likely be required [3, 4].

It should be noted that our analyses are limited to allergic and autoimmune diseases. Therefore, further studies of other diseases are needed to examine if the findings are scalable.

## Conclusions

We present a scalable framework to model and prioritize between dynamic changes in digital twins, on cellulome- and genome-wide scales. The importance lies in that each allergic and inflammatory disease may involve thousands of differentially expressed genes across multiple cell types, which vary at different disease stages. Therefore, prioritization of biomarkers and drug targets is formidable challenges. The novelties lie in that organization and analysis of cellulome- and genome-wide data in digital twins have recently been described as intractable. We propose that our framework allows organization and prioritization of UR genes for biomarker and drug discovery. This may have far-reaching clinical implications, including identification of biomarkers for personalized treatment, new drug candidates, and time-dependent personalized prescriptions of drug combinations.

## Supplementary Information

**Additional file 1.** Information about participants in the scRNA-seq SAR analyses.

**Additional file 2.** References used for cell type identification using RCA.

**Additional file 3: Fig. S1.** Diversity of Th2 cytokines in supernatants and in sera from allergen stimulated PBMC between allergic individuals at different time points. For each individual, were retrieved out of pollen season. The expression level of (A) IL-4, (B) IL-5, and (C) IL-13 in supernatant of each allergic individual. (D) the expression level of Th2 cytokines in sera in each allergic individual. **Fig. S2.** PCA plots from cell type identification. The cell type identification of (A) allergic patients, separating cells into B cells, Monocytes, Dendritic cells and T/NK cells, (B) allergic patients, separating the T/NK cells from (A) into CD4+ T cells, CD8+ T cells, and NK cells, C) allergic patients, separating the CD4+ T cells from (B) into Th1-, Th2-, Th17-, and regulatory T cells, (D) healthy controls, separating cells into B cells, Monocytes, Dendritic cells and T/NK cells, (E) healthy controls, separating the T/NK cells from (D) into CD4+ T cells, CD8+ T cells, and NK cells, and (F) healthy controls, separating the CD4+ T cells from (E) into Th1-, Th2-, Th17-, and regulatory T cells, as described in Materials and Methods. **Fig. S3.** Cell type proportions in the different groups of allergen-stimulated and diluent-stimulated samples from non-allergic and allergic individuals at the different time points. **Fig. S4.** The expression level of Th1/Th2 cytokines in different cells and time points. Dot plot showing fold changes of key Th1/Th2 cytokines in scRNA-seq. Only the statistically significant changes (*P-*value < 0.05) are presented. . **Fig. S5.** Network represents the interactions between differentially expressed genes (DEGs) from a microarray analysis of skins from AD patients and healthy controls. The red-colored nodes indicates that the gene expression level is higher in patients than in healthy controls. The green-colored nodes indicates that the gene expression level is lower in AD patients than in healthy controls.. **Fig. S6.** Multicellular network models (MNM) from scRNA-seq data of allergen- vs. diluent-stimulated PBMC in (A) healthy controls and (B) SAR patients. **Supplementary note 1**.

**Additional file 4.** Number of cells captured after filtering of the data, and quality parameters (number of reads and transcripts), for healthy controls and allergic patients, respectively.

**Additional file 5.** Significant pathways enriched in the scRNA-seq SAR data, in different cell types and at different time points.

**Additional file 6.** Drug targets identified for each separate cell type and time point.

**Additional file 7 **Results from the differential expression analysis of key genes in scRNAseq SAR data, between allergen- vs diluent-stimulated cells in patients and controls, respectively, and between allergen-stimulated cells in patients vs controls. Only statistically significant changes are presented (*P*- value < 0.05).

**Additional file 8. **Prioritization of all identified URs. The z score indicates the activation state of an upstream regulator. The farther the activation z score is from zero, the more likely it is that the direction of differential expression of the target genes is consistent with the regulator being in either an “activated” or an “inhibited” state. The URs (columns) are presented in ranked order, from left to rights (decreasing), based on the number of cell types that each UR is predicted to regulate at different time points (|z-score| ≥ 2 and *P*-value < 0.05). The data is illustrated in Fig. 7D.

**Additional file 9. **Significantly enriched pathways (*P*-value < 0.05) of differentially expressed downstream target genes of *PDGFB* in scRNAseq of SAR.

**Additional file 10. **Significantly enriched pathways (*P*-value < 0.05) of differentially expressed downstream target genes of *IFNA* in scRNAseq of SAR.

**Additional file 11. **Significantly enriched pathways (*P*-value < 0.05) of differentially expressed downstream target genes of *CCL5* in scRNAseq of SAR.

**Additional file 12.** Results for bulk data (AD) including DEGs, pathway enrichment analysis, drug target results, and URs in lesional and non-lesional samples compared to healthy control.

**Additional file 13.** Results for bulk data (UC) including DEGs, pathway enrichment analysis, drug targets results, and URs in lesional and non-lesional samples compared to healthy control.

**Additional file 14.** Results for bulk data (CD) including DEGs, pathway enrichment analysis, drug targets results, and URs in lesional and non-lesional samples compared to healthy control.

**Additional file 15.** Results for scRNA-seq data (CD) including drug targets and URs in lesional and non-lesional samples compared to healthy control.

**Additional file 16.** Results for scRNA-seq data (UC) including drug targets and URs in lesional and non-lesional samples compared to healthy control.

**Additional file 17.** GWAS enrichment results and results from pathway analysis of those GWAS genes, for lesional and non-lesional DEGs and URs, in AD, UC, and CD.

**Additional file 18.** Results from cell type identification and differential expression analysis after using different values of k during knn-smoothing.

## Data Availability

The single-cell raw and processed data generated in this study is publicly available on GEO, with accession number GSE180697 [66]. The codes generated during this study are publicly available at https://github.com/SDTC-CPMed/DigiTwin_framework [67]. The datasets used for reference construction are publicly available in BioGPS (biogps.org/downloads/), Human U133A/GNF1H Gene Atlas [34], and upon request from the authors of [20]. The bulk cell-profiling datasets for meta-analysis can be found on GEO, GSE75214 [40], and GSE32924 [41]. The DEGs based on single-cell data for meta-analysis were downloaded from [46], and the processed single cell data for meta-analysis can be found on EMBL-EBI, E-MTAB-8901 [47].

## Abbreviations

FDA: Food and Drug Administration
UR: Upstream regulator
TNF: Tumor necrosis factor-alpha
IL: Interleukin
scRNA-seq: Single-cell RNA-sequencing
DEGs: Differentially expressed genes
MNMs: Multicellular network models
PBMC: Peripheral blood mononuclear cells
HIV: Human immunodeficiency viruses
SAR: Seasonal allergic rhinitis
Th2: Type2 T helper
IFN-γ: Interferon gamma
PDGFB: Platelet-derived growth factor B
BPE: Birch pollen extract
RCA: Reference Component Analysis
NT: Naïve T
Treg: Regulatory T
Ed: Euclidian distance
IPA: Ingenuity pathway analysis
AHFS: American Hospital Formulary Service
IFNAR2: IFN alpha and beta receptor subunit 2
PRL: Prolactin
CCL5: C–C motif chemokine ligand 5
IL1RN: IL1 receptor antagonist
VEGF: Vascular endothelial growth factor A
AD: Atopic dermatitis
UC: Ulcerative colitis
NCBI: National Center for Biotechnology Information
CD: Crohn’s disease
CSF2: Colony-stimulating factor 2
GWAS: Genome-Wide Association Study
TREM1: Triggering receptor expressed on myeloid cells 1
RT: Room temperature
FBS: Fetal bovine serum
SI: Stimulation indices
KNN: K-nearest neighbor
NK: Natural killer
