# Supplementary material for: A dynamic single cell-based framework for digital twins to prioritize disease genes and drug targets
Source: Genome Med. 2022 May 6;14:48. doi: 10.1186/s13073-022-01048-4 (PMC9074288; doi:10.1186/s13073-022-01048-4)
Supplement: Supplementary file 3 — Additional file 3: Fig. S1. Diversity of Th2 cytokines in supernatants and in sera from allergen stimulated PBMC between allergic individuals at different time points. For each individual, were retrieved out of pollen season. The expression level of (A) IL-4, (B) IL-5, and (C) IL-13 in supernatant of each allergic individual. (D) the expression level of Th2 cytokines in sera in each allergic individual. Fig. S2. PCA plots from cell type identification. The cell type identification of (A) allergic patients, separating cells into B cells, Monocytes, Dendritic cells and T/NK cells, (B) allergic patients, separating the T/NK cells from (A) into CD4+ T cells, CD8+ T cells, and NK cells, C) allergic patients, separating the CD4+ T cells from (B) into Th1-, Th2-, Th17-, and regulatory T cells, (D) healthy controls, separating cells into B cells, Monocytes, Dendritic cells and T/NK cells, (E) healthy controls, separating the T/NK cells from (D) into CD4+ T cells, CD8+ T cells, and NK cells, and (F) healthy controls, separating the CD4+ T cells from (E) into Th1-, Th2-, Th17-, and regulatory T cells, as described in Materials and Methods. Fig. S3. Cell type proportions in the different groups of allergen-stimulated and diluent-stimulated samples from non-allergic and allergic individuals at the different time points. Fig. S4. The expression level of Th1/Th2 cytokines in different cells and time points. Dot plot showing fold changes of key Th1/Th2 cytokines in scRNA-seq. Only the statistically significant changes (P-value < 0.05) are presented. . Fig. S5. Network represents the interactions between differentially expressed genes (DEGs) from a microarray analysis of skins from AD patients and healthy controls. The red-colored nodes indicates that the gene expression level is higher in patients than in healthy controls. The green-colored nodes indicates that the gene expression level is lower in AD patients than in healthy controls.. Fig. S6. Multicellular network models (MNM) [file 13073_2022_1048_MOESM3_ESM.docx]

Additional file 3

Title: A dynamic single cell-based framework for digital twins to prioritize disease genes and drug targets

**Authors:** Xinxiu Li^1,†^,  Eun Jung Lee^1,2,†^, Sandra Lilja^1,†^, Joseph Loscalzo^3,4^, Samuel Schäfer^1^, Martin Smelik^1^, Maria Regina Strobl^5^, Oleg Sysoev^6, ‡^, Hui Wang^7^, Huan Zhang^1^, Yelin Zhao^1^, Danuta R. Gawel^1, †,‡^, Barbara Bohle^5, †,‡^, Mikael Benson^1,8,9,*, †,‡^

**Affiliations:**

^1^Centre for Personalized Medicine, Linköping University; Linköping, Sweden.

^2^Department of Otorhinolaryngology, Yonsei University Wonju College of Medicine; Wonju, Korea.

^3^Department of Medicine, Brigham and Women's Hospital and Harvard Medical School; Boston, MA, USA^.^

^4^Channing Division of Network Medicine, Brigham and Women's Hospital, Harvard Medical School; Boston, MA, USA.

^5^Department of Pathophysiology and Allergy Research, Center for Pathophysiology, Infectiology and Immunology, Medical University of Vienna; Vienna, Austria.

^6^Division of Statistics and Machine Learning, Department of Computer and Information Science, Linkoping University; Linköping, Sweden.

^7^Jiangsu Key Laboratory of Immunity and Metabolism, Department of Pathogenic Biology and Immunology, Xuzhou Medical University; Jiangsu, China.

^8^Crown princess Victoria Children’s Hospital, Linköping University Hospital; Linköping, Sweden.

^9^Division of ENT Diseases, Department of Clinical Sciences, Intervention and Technology, Karolinska Institutet; Stockholm, Sweden.

^*^Corresponding author. Email: [mikael.benson@liu.se](mailto:mikael.benson@liu.se).

^†^ These authors contributed equally to this work.

^‡^ These authors jointly supervised these studies.

Supplementary Figures


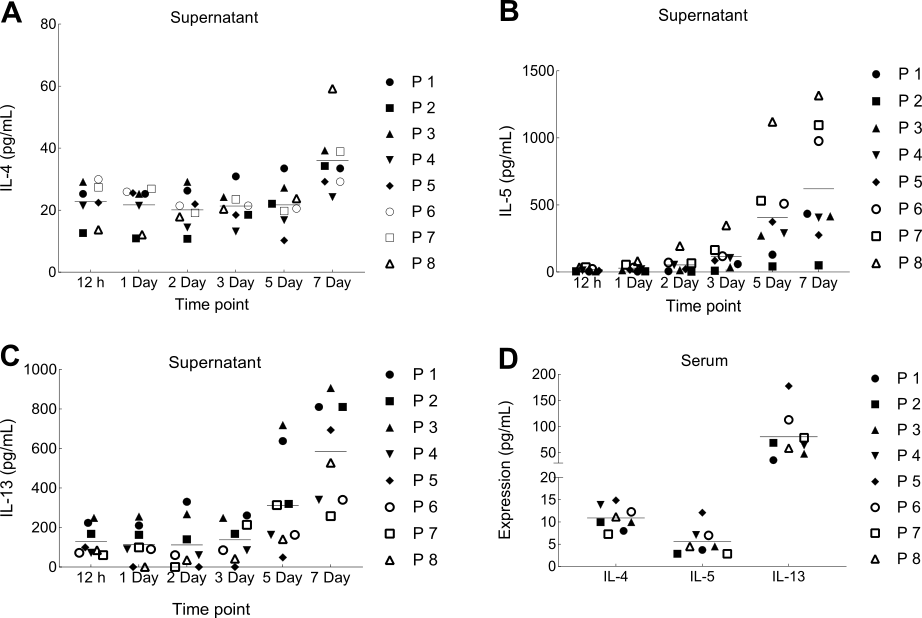
 **Fig. S1. Diversity of Th2 cytokines in supernatants and in sera from allergen stimulated PBMC between allergic individuals at different time points.** For each individual, were retrieved out of pollen season. The expression level of **(A)** IL-4, **(B)** IL-5, and **(C)** IL-13 in supernatant of each allergic individual. **(D)** the expression level of Th2 cytokines in sera in each allergic individual.


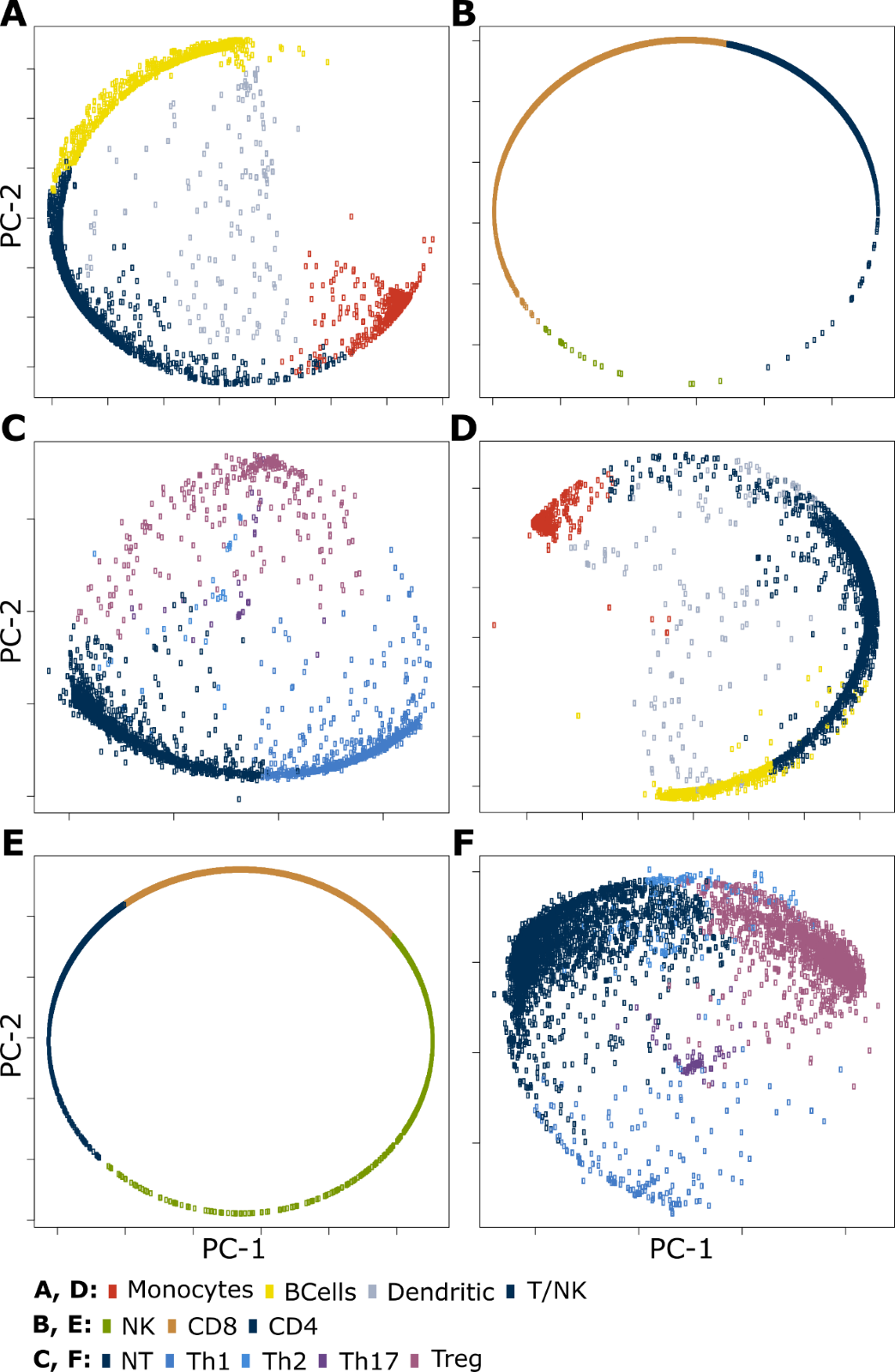


**Fig. S2.** **PCA plots from cell type identification.** The cell type identification of **(A)** allergic patients, separating cells into B cells, Monocytes, Dendritic cells and T/NK cells, **(B)** allergic patients, separating the T/NK cells from (A) into CD4+ T cells, CD8+ T cells, and NK cells, **C)** allergic patients, separating the CD4+ T cells from (B) into Th1-, Th2-, Th17-, and regulatory T cells, **(D)** healthy controls, separating cells into B cells, Monocytes, Dendritic cells and T/NK cells, **(E)** healthy controls, separating the T/NK cells from (D) into CD4+ T cells, CD8+ T cells, and NK cells, and **(F)** healthy controls, separating the CD4+ T cells from (E) into Th1-, Th2-, Th17-, and regulatory T cells, as described in Materials and Methods.


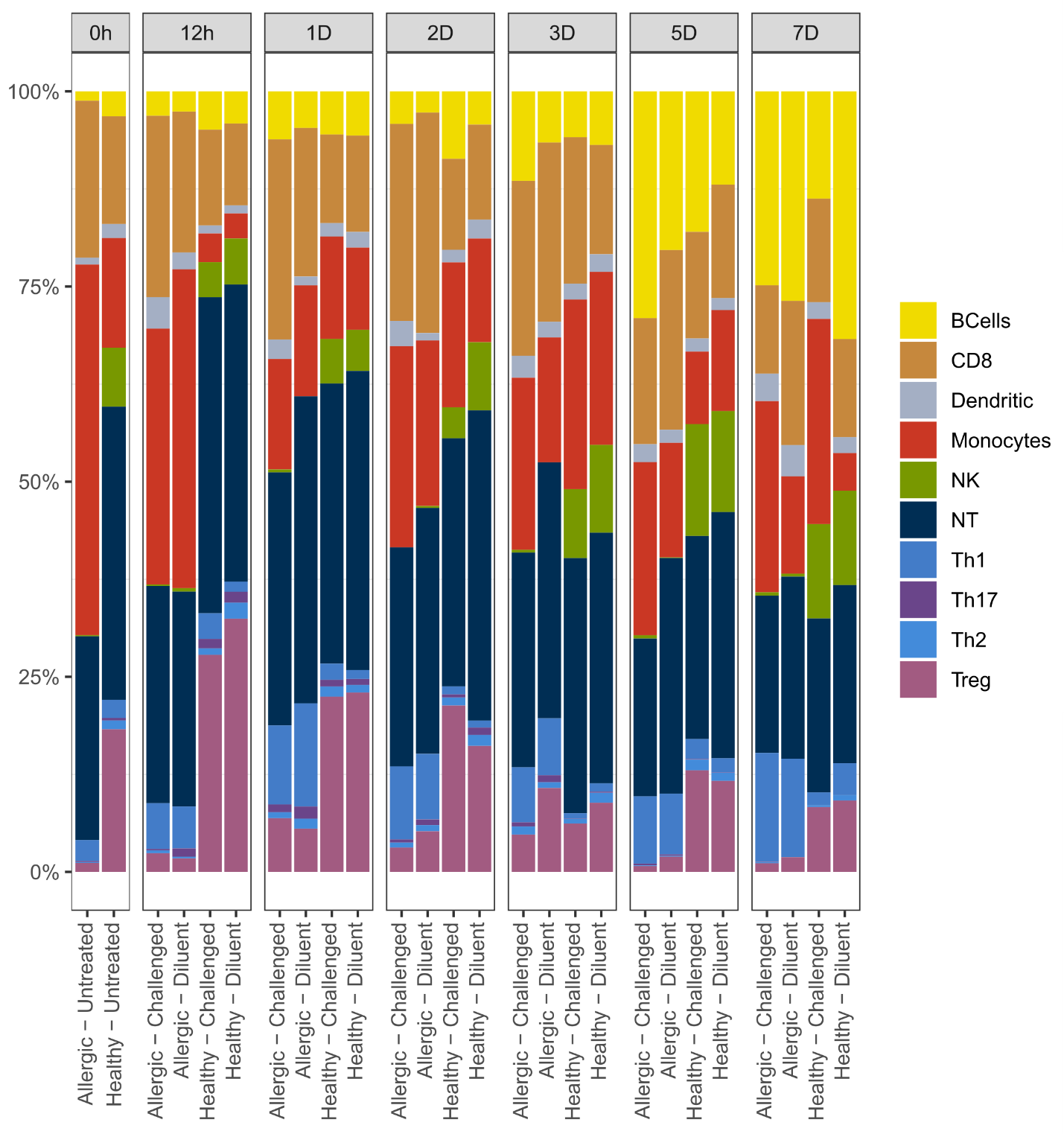
**Fig. S3.** Cell type proportions in the different groups of allergen-stimulated and diluent-stimulated samples from non-allergic and allergic individuals at the different time points.


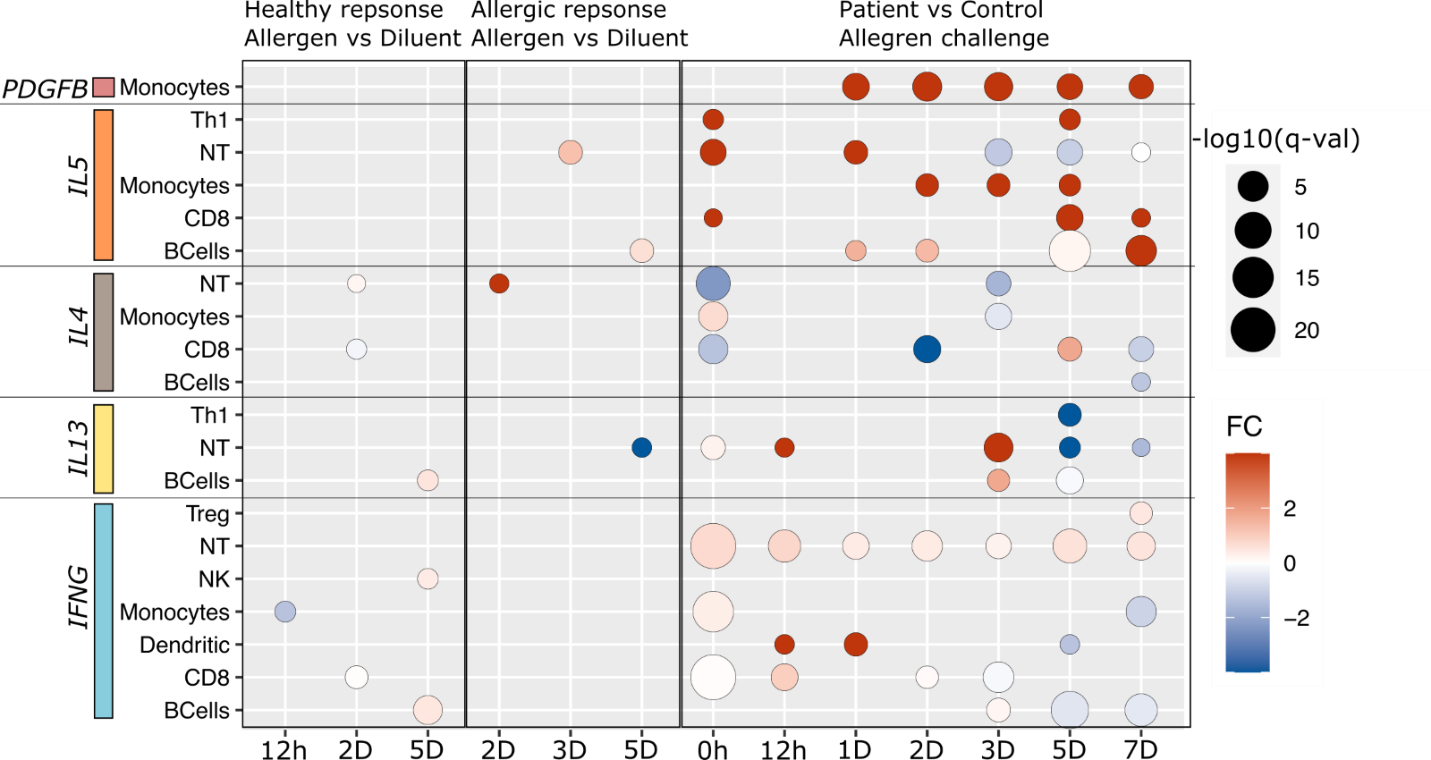


**Fig. S4.** **The expression level of Th1/Th2 cytokines in different cells and time points.** Dot plot showing fold changes of key Th1/Th2 cytokines in scRNA-seq. Only the statistically significant changes (*P-*value < 0.05) are presented.


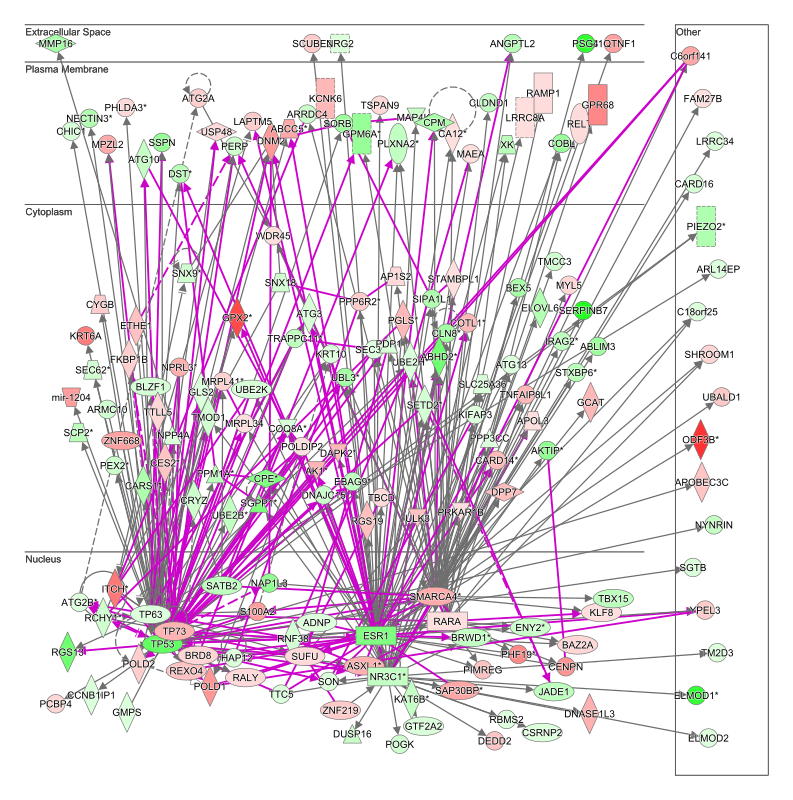


**Fig. S5. Network represents the interactions between differentially expressed genes (DEGs) from a microarray analysis of skins from AD patients and healthy controls.** The red-colored nodes indicates that the gene expression level is higher in patients than in healthy controls. The green-colored nodes indicates that the gene expression level is lower in AD patients than in healthy controls.


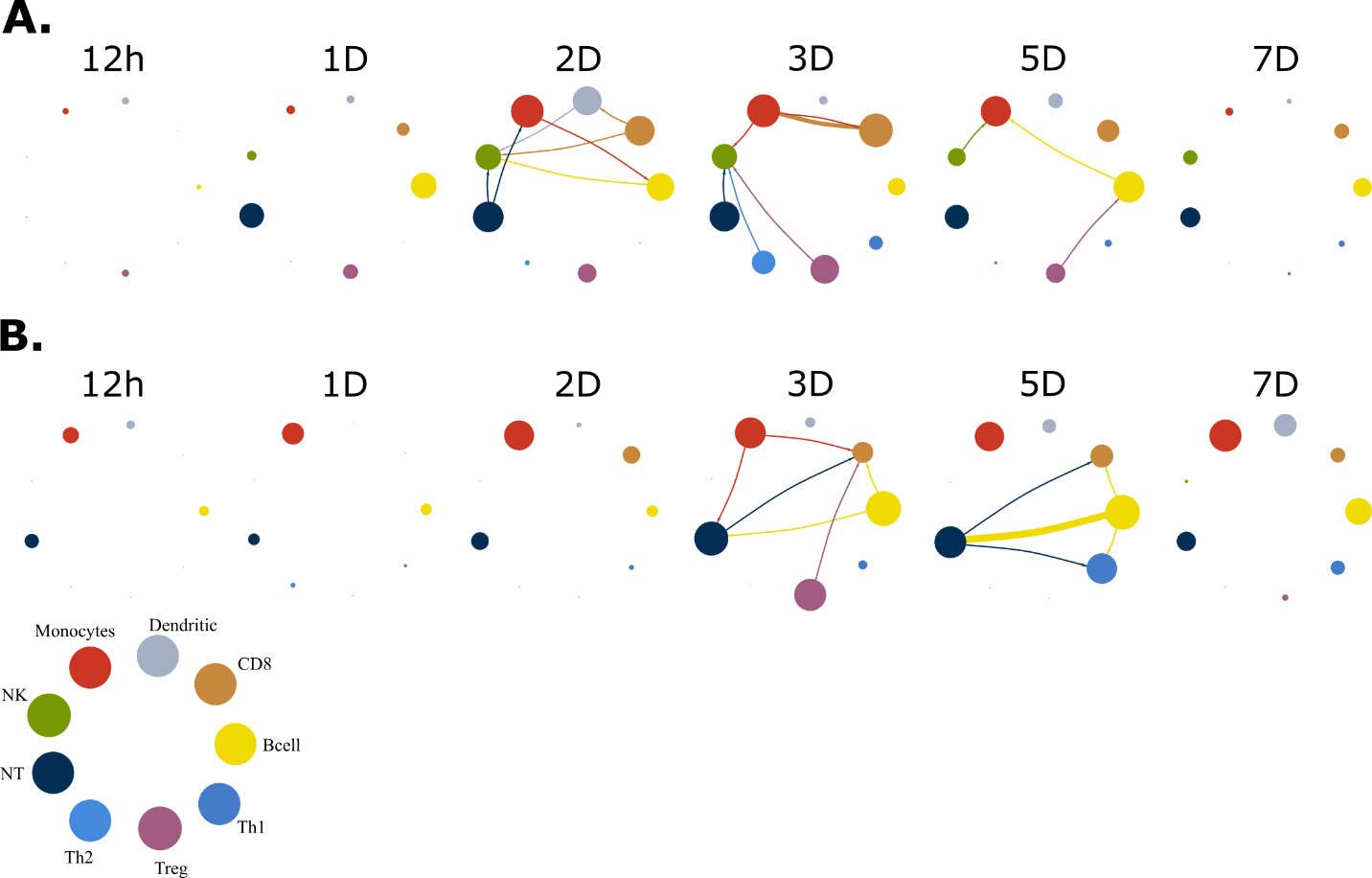


**Fig. S6 . Multicellular network models (MNM) from scRNA-seq data of allergen- vs. diluent-stimulated PBMC in (A) healthy controls and (B) SAR patients.**

Supplementary Note 1

This is a comment regarding the shape of Principal Component visualization of how each single cell transcriptome correlated with cell type-specific bulk transcriptomes (Figure 3A). The main reasons of the observed atypical shape of data points are 1) that the data used for the visualization are normalized by the RCA approach by applying z-score transformation to each observation (i.e. subtracting its mean and dividing by its standard deviation) which allocates the data on the surface of a unit sphere in the multidimensional space 2) each observation is a vector of correlations to the bulk gene expressions, rather than single cell gene expressions themselves. Reason 1) is the main reason of observing “circular shapes” in the projections.
