# Supplementary material for: A dynamic single cell-based framework for digital twins to prioritize disease genes and drug targets
Source: Genome Med. 2022 May 6;14:48. doi: 10.1186/s13073-022-01048-4 (PMC9074288; doi:10.1186/s13073-022-01048-4)
Supplement: Supplementary file 18 — Additional file 18. Results from cell type identification and differential expression analysis after using different values of k during knn-smoothing. [file 13073_2022_1048_MOESM18_ESM.docx]

K-nearest neighbor (KNN)-smoothing with python (v3.7.4) was applied to reduce the effect of dropouts and other noise. The choice of k affects the downstream analyses, such as cell typing and differential expression analyses which in their turn affect MNM resolution. Cell typing and differential expression analysis (allergen stimulated vs diluent stimulated) was done as in the main methods, with some modifications; R version 4.0.4 was used, and the data input to the differential expression analysis script (pre-DEG_analysis.R,  [https://github.com/SDTC-CPMed/DigiTwin_framework](https://eur01.safelinks.protection.outlook.com/?url=https%3A%2F%2Fgithub.com%2FSDTC-CPMed%2FDigiTwin_framework&data=04%7C01%7Cxinxiu.li%40liu.se%7C8b2f4b36a8bb4af1370408d94c2c11c1%7C913f18ec7f264c5fa816784fe9a58edd%7C0%7C0%7C637624574426324550%7CUnknown%7CTWFpbGZsb3d8eyJWIjoiMC4wLjAwMDAiLCJQIjoiV2luMzIiLCJBTiI6Ik1haWwiLCJXVCI6Mn0%3D%7C1000&sdata=z%2Fl4TrmB09yOOs07wPFoV8mfv3Qm6m4Nfx0IpUwOKYY%3D&reserved=0)) contained gene names as ‘Symbols’ instead of ‘ENTREZ’ IDs, so that later translation between the gene naming systems was omitted. The results in Additional table 1 and 2 demonstrate that both number of cells per cell type and the number of differentially expressed genes can be greatly affected by the choice of k so an appropriate choice of k is important. We have followed biological principles presented in [1] to choose appropriate values of k. More specifically, k values were chosen according to the expected proportion of the least common cell type in the reference (CD3+CD56+ cells) which is expected to be 0.8+/-0.7%. This resulted in k = 14 and k = 21 for samples from the allergic and healthy individuals, respectively.

The number of cells of a cell type is another important factor that affects the resolution of MNMs. Cell types having very few cells may lead to the lack of statistical significance in DEG hypothesis testing leading to few genes being detected as significantly differentially expressed. This limitation of the approach is demonstrated by Additional table 2 where Dendritic cells have zero DEGs for larger k, which is a direct effect of over-smoothing as rare cell types are lost. This kind of problem can also be observed for the values of k that we finally select, as we generally detect few or no DEGs in the rare cell types (Fig. 7C and Additional file 3: Supplementary Fig. 3).

**Additional table 1.** Number of cells identified for each cell type in healthy samples after performing knn-smoothing using different values of k.

| **celltype** | **K10** | **K20** | **K30** | **K40** | **K50** |
| --- | --- | --- | --- | --- | --- |
| **BCells** | 1,034 | 1,031 | 1,437 | 1,447 | 1,509 |
| **CD8** | 441 | 1,790 | 627 | 233 | 505 |
| **Dendritic** | 229 | 112 | 6 | 0 | 7 |
| **Monocytes** | 2,889 | 2,788 | 2,808 | 2,770 | 2,798 |
| **NK** | 276 | 383 | 421 | 456 | 380 |
| **NT** | 5,196 | 8,387 | 10,495 | 13,243 | 12,580 |
| **Th1** | 2,207 | 1,320 | 1,715 | 680 | 1,252 |
| **Th17** | 448 | 226 | 143 | 58 | 26 |
| **Th2** | 437 | 36 | 146 | 122 | 141 |
| **Treg** | 3,236 | 2,805 | 2,149 | 1,278 | 1,330 |

**Additional table 2.** Number of DEGs identified between allergen stimulated and diluent stimulated samples at day 3, for each cell type in healthy samples after performing knn-smoothing using different values of k.

| **celltype** | **K10** | **K20** | **K30** | **K40** | **K50** |
| --- | --- | --- | --- | --- | --- |
| **BCells** | 186 | 459 | 1,836 | 2,011 | 4,255 |
| **CD8** | 1,863 | 6,271 | 5,194 | 657 | 6,289 |
| **Dendritic** | 6 | 2 | 0 | 0 | 0 |
| **Monocytes** | 876 | 3,295 | 5,549 | 5,729 | 6,902 |
| **NK** | 2 | 36 | 27 | 97 | 31 |
| **NT** | 2,138 | 3,427 | 7,610 | 9,972 | 9,977 |
| **Th1** | 1,169 | 23 | 515 | 34 | 390 |
| **Th17** | 1,505 | 0 | 2 | 0 | 0 |
| **Th2** | 13 | 0 | 1 | 0 | 0 |
| **Treg** | 371 | 1,533 | 36 | 3 | 15 |

References

1. Blood [Internet]. Miltenyti Biotec. Available from: https://www.miltenyibiotec.com/US-en/resources/macs-handbook/human-cells-and-organs/human-cell-sources/blood-human.html
